# *Dry* Selection and *Wet* Evaluation of New 1,2-Disubstituted Nitroindazolin-3-One Derivatives as Promising Agents Against *Trypanosoma cruzi*

**DOI:** 10.3390/ph19091473

**Published:** 2026-09-17

**Authors:** Juan A. Castillo-Garit, Josue Pozo-Martinez, Esteban Rocha-Valderrama, Cristian Rojas-Peña, Karen Acosta-Quiroga, Vicente J. Arán, Claudio Olea-Azar, Gerardo M. Casañola-Martin, Bakhtiyor Rasulev, Francisco Torrens, Facundo Pérez-Giménez, Mauricio Moncada-Basualto

**Affiliations:** 1 Instituto Universitario de Investigación y Desarrollo Tecnológico (IDT), Universidad Tecnológica Metropolitana, Ignacio Valdivieso 2409, San Joaquín, Santiago 8940577, Chile; esteban.rocha@ug.uchile.cl (E.R.-V.); cristian.rojas.p@ug.uchile.cl (C.R.-P.); karenacosta@ug.uchile.cl (K.A.-Q.); 2 Centro de Investigación, Desarrollo e Innovación de Productos Bioactivos (CinBio), Escuela de Química y Farmacia, Facultad de Farmacia, Universidad de Valparaíso, Av. Gran Bretaña 1093, Valparaiso 2360102, Chile; josue.pozo@uv.cl; 3 Laboratorio de Química-Médica, Facultad de Ciencia y Tecnología, Universidad del Azuay, Cuenca 010204, Ecuador; 4 Laboratorio de Radicales Libres y Antioxidantes, Facultad de Ciencias Químicas y Farmacéuticas, Universidad de Chile, Santiago 8330015, Chile; colea@uchile.cl; 5 Instituto de Química Médica (CSIC), Juan de la Cierva 3, 28006 Madrid, Spain; uvejotaran@gmail.com; 6 Department of Coatings and Polymeric Materials, North Dakota State University, Fargo, ND 58102, USA; gerardo.casanolamart@ndsu.edu (G.M.C.-M.); bakhtiyor.rasulev@ndsu.edu (B.R.); 7 Institut Universitari de Ciència Molecular, Universitat de Valencia, 46010 Valencia, Spain; francisco.torrens@uv.es; 8 Research Unit of Radiopharmacy and Stability Drugs, Universidad de Valencia, 46010 Valencia, Spain; facundo.perez@uv.es

**Keywords:** 1,2-disubstituted nitroindazolin-3-one, Chagas disease, oxidative stress, random forest, trypanocidal activity

## Abstract

**Background/Objectives:** Chagas disease is endemic to 21 Latin American countries and is a great public health problem. Current chemotherapy remains unsatisfactory; consequently, the need to search for new drugs persists. The aim of this work is to develop a machine learning computational model, which allows the identification of new chemical compounds with potential trypanosomicidal activity. **Methods:** A large dataset of 584 compounds, obtained from the Drugs for Neglected Diseases initiative, is used to develop the computational model. AlvaDesc v3.0.14 software is used to calculate the molecular descriptors, and Scikit-learn of Python to obtain the random forest. **Results:** The best random forest model shows accuracy of 82.1% for the training set and near to 79% for the test set, achieving specificity values over 84.9%, and the false alarm rate values were almost under 15% for both sets. As an experiment of virtual lead generation, the present model is finally satisfactorily applied to the virtual evaluation of a series of 1,2-disubstituted nitroindazolin-3-ones obtained a good agreement between the predicted activity and the experimental assays performed. Compounds **1c** and **2c** stood out as the most active in the series, with half-maximal inhibitory concentration (IC_50_) values of 71.3 and 40.9 μM, respectively. Mechanistic analyses suggest that the presence of the nitro group may promote the generation of reactive oxygen species through enzymatic redox activation, potentially involving *T. cruzi* nitroreductases (TcNTRs), leading to oxidative-stress-mediated parasite death. For the most active compounds, the data are consistent with intracellular hydroxyl radical generation through enzymatic redox processes. **Conclusions:** Even though none of them resulted more active than nifurtimox, the current results constitute a step forward in the search for efficient ways to discover new lead antitrypanosomals.

## 1. Introduction

Chagas disease (CD), a neglected tropical disease also known as American trypanosomiasis, is a potentially life-threatening illness caused by the protozoan parasite *Trypanosoma cruzi* [1]. More than a century has passed since, in 1909, the Brazilian scientist Carlos Chagas discovered the intracellular protozoan parasite *T. cruzi* as the causative agent of the disease that bears his name [2]. Since then, CD has gained importance, with several socioeconomic, environmental, and public health problems [3]. About eight million people globally, including an estimated 280,000 in the United States, have this disease, usually without knowing it [4]. An estimated 10,000 people die from Chagas disease every year, and over 100 million people are at risk of acquiring the disease [5].

The main transmission mechanism for CD in endemic areas is mediated by triatomine vectors. Other mechanisms of infection that are important, especially in nonendemic areas, include blood transfusion, organ transplantation, oral ingestion, laboratory accidents, vertically from mother to child, or shared intravenous needles [6]. The movement of people living with Chagas disease from rural areas to cities and other countries has changed where we find Chagas disease. In countries like the United States, where Chagas disease is present but is not regularly spread by triatomine bugs, it is important to recognize infection and to prevent its spread through blood transfusion, organ transplants, and from pregnant women to their babies [4]. In the transmission mediated by triatomine bugs (members of the Reduviidae bug family), the metacyclic trypomastigotes (infective form *T. cruzi*) enter humans via triatomine excretion: triatomines have a habit of defecating while feeding on blood, and the parasite enters the host via the mucous membranes or breaks in the skin triggered by scratching while being bitten [6].

The clinical course of Chagas disease usually comprises an acute phase and a chronic phase. First, in the acute phase during the first and second weeks after infection, parasites invade host cells and replicate. The host cells then rupture, and the parasites undergo hematogenous spread, during which they are visible on microscopic examination. In spite of the large numbers of parasites in the blood, infected people seldom have severe symptoms. Typically, they experience mild fever and malaise and, if examined, show some enlargement of the liver and the spleen [7,8]. In general, the acute phase lasts 4–8 weeks, and parasitemia decreases substantially from 90 days onwards [9]. Cellular immunity develops, symptoms improve, and the parasites are cleared from the blood. In the other phase (chronic), parasites remain in the muscles of the gastrointestinal tract and the myocardium for years, evoking a chronic immune response. About 30–40% of people with Chagas disease will see it evolve to chronic symptomatic forms within 10–30 years, such as the Chagas heart disease, the most prevalent symptomatic form, and digestive forms, characterized by a megaesophagus or megacolon (or both) [1].

The World Health Organisation recognized CD as a neglected tropical disease 20 years ago in 2005. This helped to gain a greater recognition of the disease and combated misinformation, the lack of social demand, and the weak political commitment to solve problems related to CD (such as insufficient scientific research, detection, and comprehensive care, including diagnosis, treatment, medicine presentations, social aspects, information, education, and communication tools) [3,10]. A public health strategy was conducted for vectorial control, in which the spraying of poor-quality housing with insecticide had a significant impact on breaking transmission cycles in some areas. However, eradication by this route is unlikely to be feasible [11]. Then, chemotherapy remains as the principal solution, but current chemotherapy remains unsatisfactory, and the available drugs are benznidazole (BZN) and nifurtimox (NFX) [12,13]. These compounds, originally registered for treatment of acute *T. cruzi* infections, were introduced in the market more than 50 years ago [13,14] and remain, to this day, the only available drugs for the specific treatment of Chagas disease [15]. Moreover, both drugs have important adverse effects such as anorexia, vomiting, peripheral neuropathy and allergic dermopathy, which can result in treatment discontinuation [15]. All these drawbacks explain the need for the search and design of new drugs against *T. cruzi* [16].

Several authors recognize that drug discovery for Chagas disease has been suffering from the lack of interest by pharmaceutical companies due to the low income of most affected people [14,17]. This explains the need for the search and design of new drugs against *T. cruzi*. In the past years, several attempts have been made to solve this problem [18,19,20,21]. Among these, predictive computational models, such as quantitative structure–activity relationships (QSARs), have been successfully applied to identify novel candidates with antitrypanosomal potential [22,23,24].

Our research group has extensive experience in developing QSAR-based classification models for the identification of bioactive compounds against various diseases [25,26,27,28,29,30,31,32]. Random forest classifiers were chosen because of their robustness to noisy and collinear descriptor spaces and their proven performance in QSAR-based virtual screening. Moreover, the availability of variable importance measures allows mechanistic interpretation of the resulting classification models. The main objective of the present study was to develop new classification models using the random forest algorithm to identify antitrypanosomal compounds within a specific chemical scaffold.

In this context, we focused on 1,2-disubstituted nitroindazolin-3-ones, selected based on well-established chemical and biological criteria. Indazolin-3-one scaffolds provide a rigid and planar heterocyclic framework with favorable electronic properties, enabling systematic modulation of steric and lipophilic features through *N*^1^,*N*^2^-disubstitution [20]. The incorporation of a nitro group was motivated by extensive evidence indicating that redox bioactivation of nitroheterocycles in *Trypanosoma cruzi* can contribute to oxidative stress and metabolic perturbations associated with antiparasitic activity [19]. Importantly, the indazole core allows for controlled variation in the nitro group position, offering a rational approach to exploring how electronic distribution and reduction propensity may influence the observed biological effects. This chemical rationale underpins the present study.

## 2. Results and Discussion

### 2.1. Development and Validation of the Computational Model

To develop the classification model, the Random Forest (RF) algorithm was applied using biological activity (Act) as the dependent variable. The best-performing RF model identified consisted of six molecular descriptors, with a maximum tree depth of 6 and a total of 31 estimators. The performance statistics of this optimized model are shown below in Table 1.

**Figure 1:**
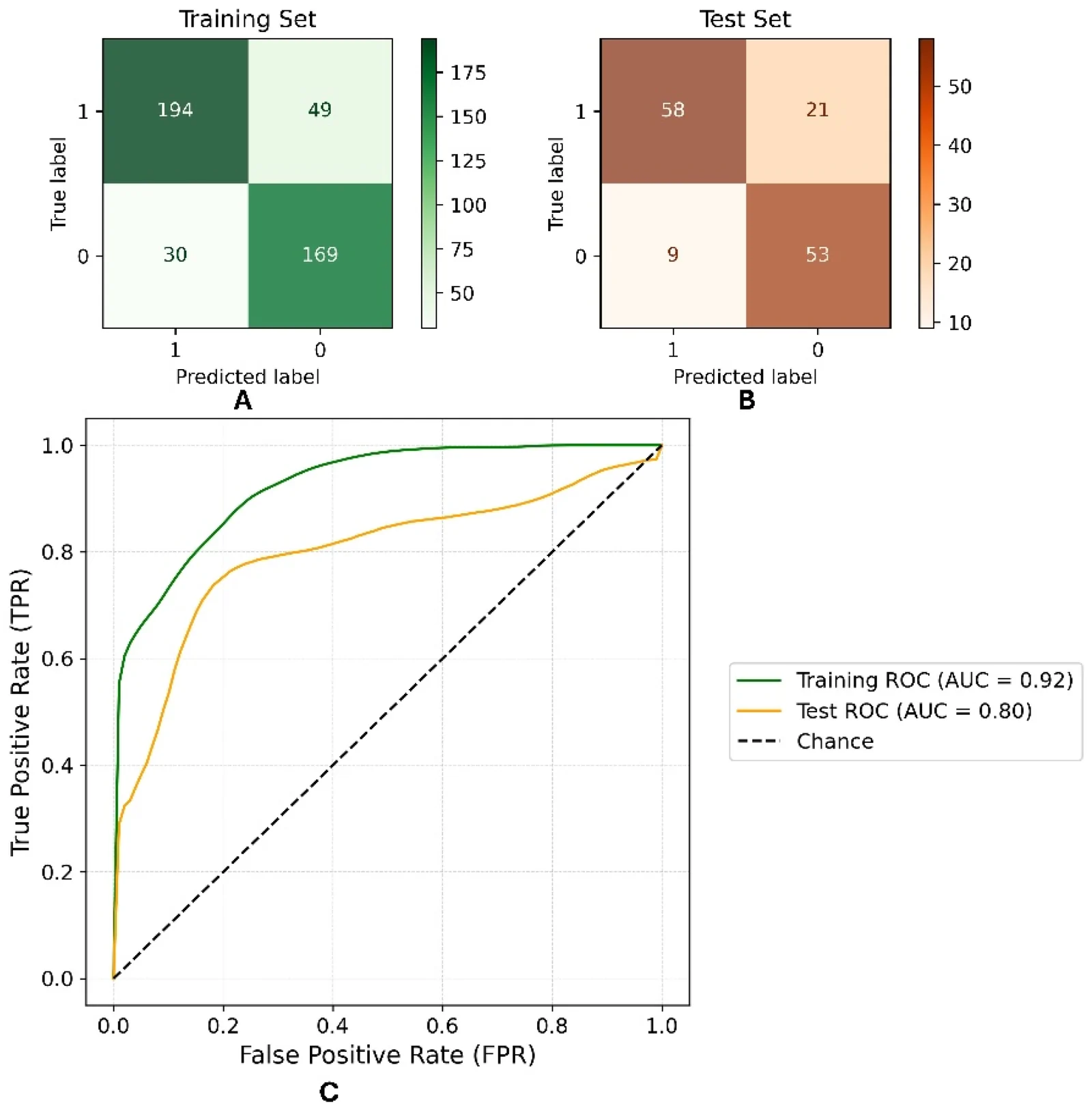
Confusion matrix for training (**A**) and prediction (**B**) sets and Receiver operating characteristic curves (**C**).

As can be seen, RF is performed with just six attributes (H-049, VE2sign_B(m), SsssCH, SpMin3_Bh(i), s3_numAroBonds, and X1A); it presents a good performance. The RF model achieved an accuracy of 82.1% on the training set and 78.7% on the test set. In this sense, the values of sensitivity were between 73.4 and 79.8%, while the specificity of the RF values for the training and test sets were 84.9% and 85.5%, respectively. The value of the false positive rate for both sets was around 15% (15.1% for training and 14.5% for test set); all these values are about the recommended value. The RF model demonstrates a strong balance between sensitivity and specificity, with good generalization to the test set maintaining low false positive rates. The confusion matrices for the training and test sets are shown in Figure 1A and Figure 1B, respectively. In addition, the Receiver Operating Characteristic (ROC) curves for both the training and test sets are shown in Figure 1C. The Areas Under the Curve (AUCs) are 0.9575 for the training set (green line) and 0.9386 for the test set (yellow line), indicating excellent classification performance in both cases. The 5-fold cross-validation yielded an accuracy of 0.645, which is consistent with the chemical and biological complexity of antitrypanosomal datasets. Considering the structural diversity and experimental variability inherent to *T. cruzi* activity data, this value indicates a statistically meaningful improvement over random classification and supports the robustness of the proposed model.

**Figure 2:**
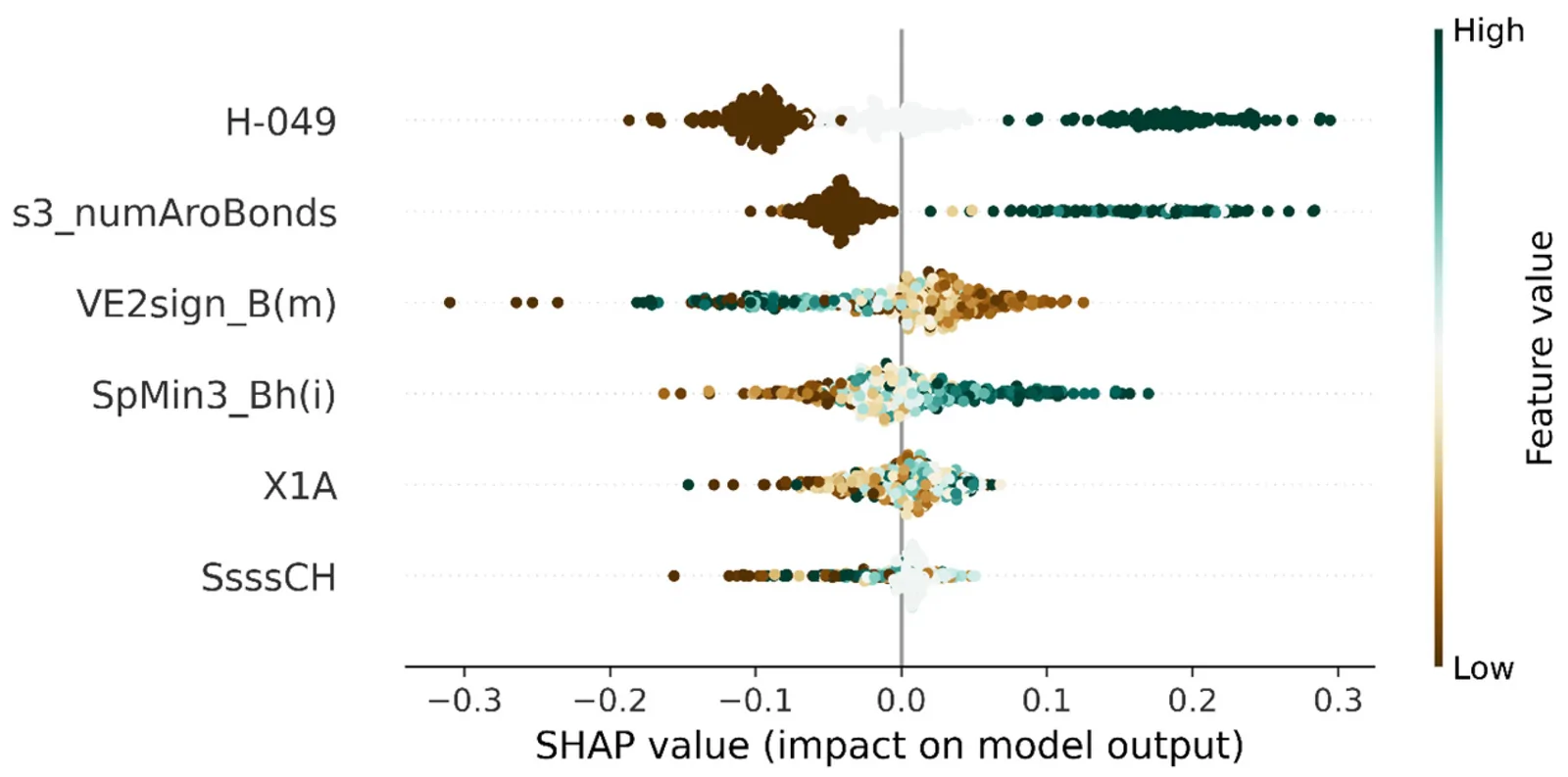
Summary plot denoting the Shapley values and impact of each descriptor.

The SHAP approach has been used in this work to interpret the RF model. Figure 2 presents the SHAP summary plot top six features; the *y*-axis sorts of features based on the average absolute SHAP values for each feature, while the *x*-axis represents SHAP values (the distribution of feature contribution on the model outcome indicates that positive values represent a positive impact of the feature on the predicted activity, whereas negative values indicate a negative impact). Green dots indicate high feature values, while brown dots represent low values. The six features are ranked according to their impact on the model, with H-049 (H attached to C3(sp^3^)/C2(sp^2^)/C3(sp^2^)/C3(sp) is a basic descriptor from the group of Atom-centered fragments) and s3_numAroBonds (number of aromatic bonds of the substituent 3 is a chirality descriptor), having the highest impact (Figure 2). The plot also indicates whether each feature contributes positively or negatively to anti-trypanosomal activity. Higher feature values leading to increased SHAP values indicate a positive correlation with activity, and vice versa.

**Figure 3:**
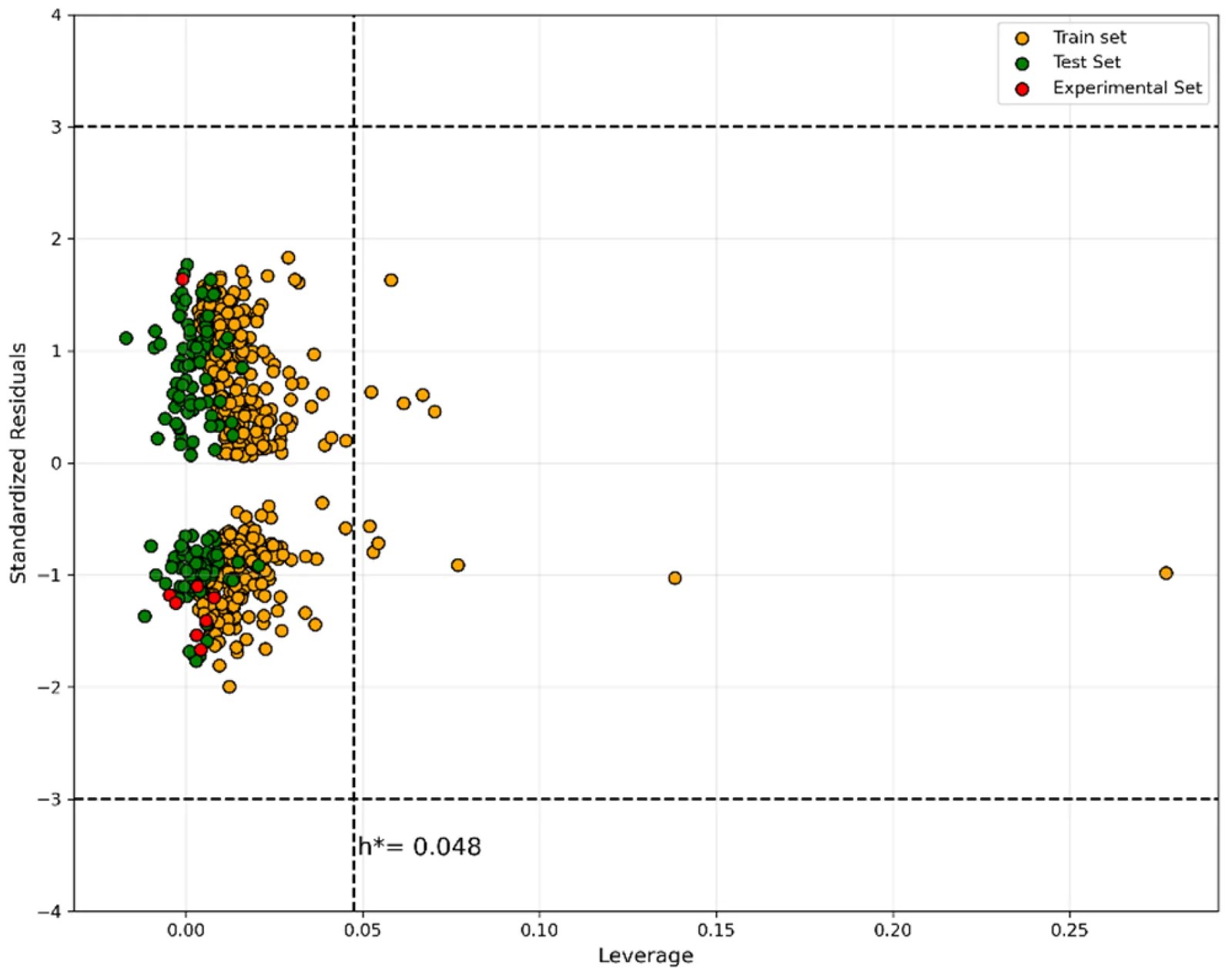
Applicability domain (AD) defined using Williams’ plot, showing leverage (hat diagonal values) versus standardized residuals. The dashed horizontal lines indicate the standardized residual limits (±3), and the vertical dashed line indicates the critical leverage (h* = 0.048), where h* denotes the leverage threshold used to identify structurally influential compounds outside the applicability domain.

Even with a robust, significant, and validated QSAR model, unreliable predictions of the modeled property for the entire universe of chemical compounds can be expected. Indeed, only predictions for compounds that fall within the AD can be considered reliable and not the extrapolation of the models. In this study, we used the approach of leverage (h), and standardized residuals described in William’s graph of the model (see Figure 3), where orange circles represent the compounds of the training and green circles represent the prediction set. As shown in the figure, most of the compounds are within the applicability domain of the model. There are few compounds with leverage values greater than the critical leverage (h* = 0.048) but showing residual within the limits.

### 2.2. Screening and Experimental Evaluation of New Anti-Trypanosomal Compounds

**Figure 4:**
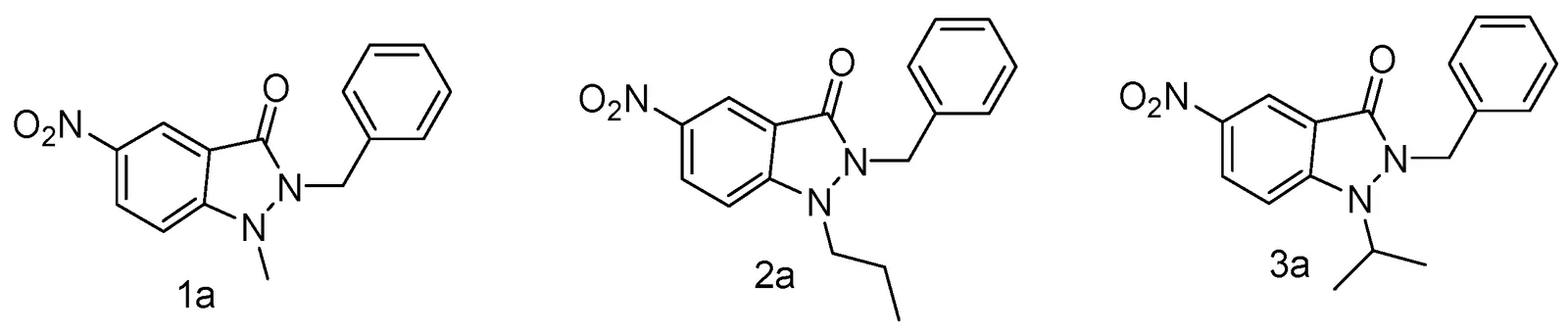
Examples of 1,2-disubstituted 5-nitroindazolin-3-ones with trypanocidal activity previously described [20,33].

Previous research has demonstrated that 1,2-disubstituted 5-nitroindazolin-3-ones exhibit notable trypanocides properties, particularly compounds **1a**–**1c** (Figure 4), which have been exhibiting effectiveness against CL-B5 epimastigote forms and low cytotoxicity [33]. Other researchers have reported good activity, in the range of 1.04 and 6.17 μM, for a group of 5-nitroindazolin-3-ones with different aromatic substituents [20].

**Figure 5:**
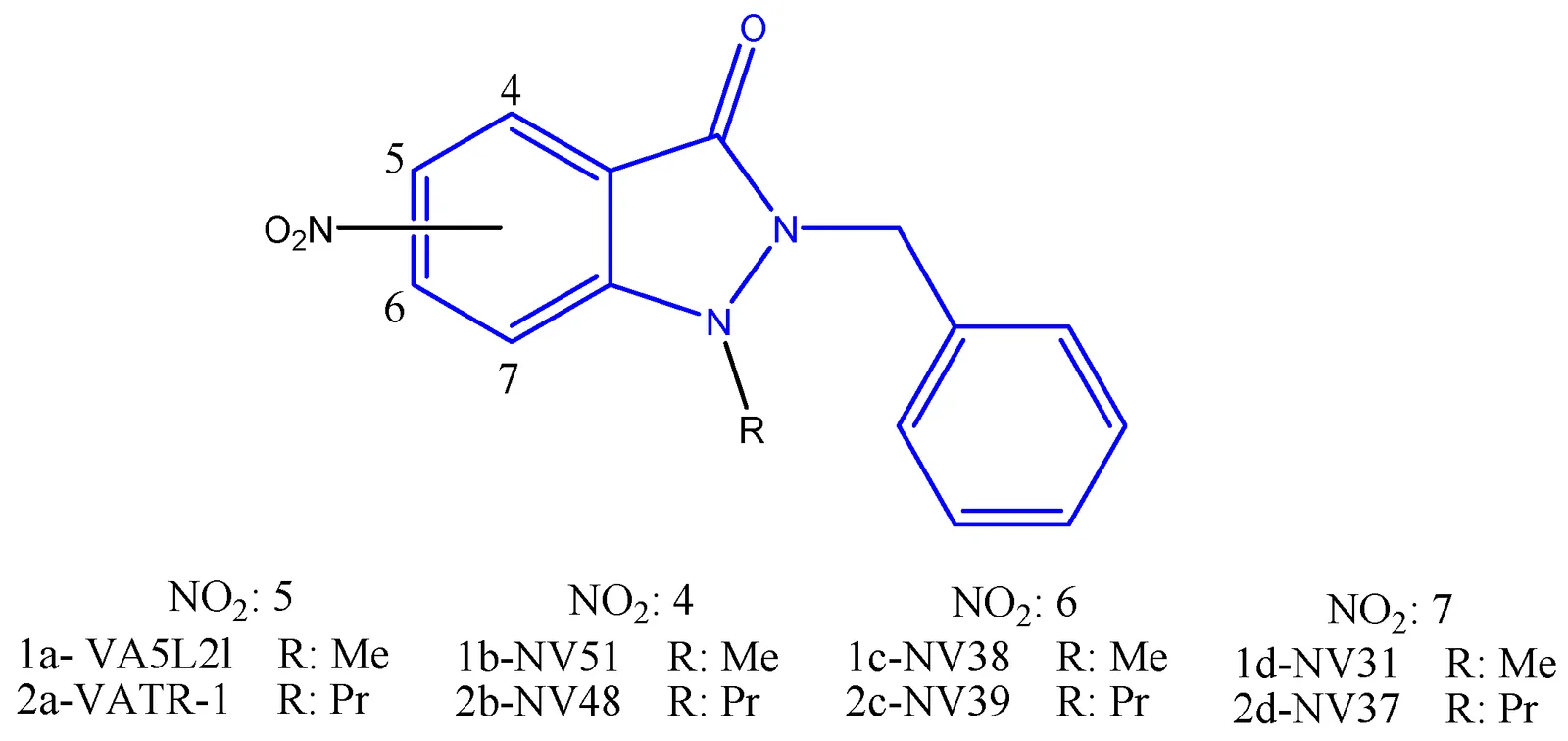
1,2-disubstituted nitroindazolin-3-ones derivatives studied in this work.

Based on these findings, a series of structurally related 1,2-disubstituted-nitroindazolin-3-ones was selected for the present study (Figure 5). The synthesis and complete chemical characterization of the **4**-, **6**-, and **7**-nitro derivatives have recently been reported by Fonseca-Berzal et al. [34]. In the present work, this compound series was used to investigate the influence of the nitro-group position on predicted antitrypanosomal activity and to further explore its biological, electrochemical, and radical-generating behavior. Given their structural similarity to the aforementioned active compounds, it was reasonable to hypothesize that they might also exhibit activity against *T. cruzi*. The aim of our study was to explore how the position of the nitro group on the indazole ring influences the biological activity of these compounds (Figure 5).

First, we evaluate the new compounds by using the previously developed computational model to predict their antitrypanosomal activity. Surprisingly, and contrary to what might be expected for this group of compounds, only one compound (2c) was identified as active by the model; this means that the IC_50_ value should be lower than 50 μM. The remaining compounds were predicted as inactive, which means that they should show IC_50_ values over 50 μM.

We found that all the compounds fall within the applicability domain of the model; therefore, the predictions can be considered reliable. In Figure 3, these compounds are represented with red circles. As shown, one compound falls into the group with predicted IC_50_ values below 50 μM, while the rest are clustered in the group with IC_50_ values above this cutoff. These initial findings prompted us to investigate further the experimental activity of the compounds and to assess whether the predicted values align with the observed biological behavior.

### 2.3. Biological Activity

To evaluate the influence of nitro-group position on trypanocidal activity, we examined the previously synthesized and fully characterized series of *N*^1^,*N*^2^-disubstituted indazolin-3-one derivatives [34], maintaining a constant core structure with either methyl or propyl at *N*^1^ and a benzyl group at *N*^2^, while varying the position of the nitro group on the aromatic ring (positions 4, 5, 6, and 7). This limited structural variability was designed to isolate the topological and electronic effects of the nitro group, aiming to identify the most promising position for future rational optimization.

*In vitro* assays showed that all tested compounds displayed some degree of activity, against the *T. cruzi* trypomastigote form (Dm28c strain), although in general, their IC_50_ values were higher than that of the reference drug nifurtimox (NFX, IC_50_ = (21.5 ± 1.4) μM). Nevertheless, compounds **1c** and **2c**, which bear the nitro group at position 6, stood out as the most active in the series, with IC_50_ values of (71.3 ± 0.9) and (40.9 ± 0.8) μM, respectively, surpassing even other previously reported derivatives bearing the nitro group at position 5, which has traditionally been considered optimal in related studies [19,20,33,35,36,37,38,39,40,41].

This result is striking, as previous studies have proposed that positioning the nitro group at position 5 in indazole derivatives may favor ROS generation through enzymatic redox activation, including mechanisms involving *Tc*NTR, which have been associated with parasite death [19]. However, in the present study, derivatives 1a and 2a (NO_2_ at position 5) exhibited significantly lower activity (IC_50_ = 189.7 and 149.6 μM), suggesting that efficient redox activation and/or spin delocalization observed by EPR does not necessarily correlate with higher trypanocidal efficacy in this series.

By contrast, compounds with the NO_2_ group at position 6 (**1c** and **2c**), not only displayed higher trypanocidal activity but also exhibited the highest selectivity indices (SI > 4.2 and >7.3), maintaining low cytotoxicity toward mammalian cells (VERO and EA.hy926). This indicates that, despite not showing extended delocalization as observed for the 5-nitro derivatives, the position 6 nitro group may allow for better enzymatic accessibility, a more favorable electronic orientation for one-electron reduction, or enhanced interaction with enzymatic redox systems previously implicated in *T. cruzi* nitroaromatic bioactivation (Table 2).

From a physicochemical standpoint, previous studies have shown that placing the nitro group at position 6 can promote a more favorable spatial orientation relative to the π-conjugated system, potentially facilitating single-electron reduction and efficient generation of reactive species, that mediate parasite death [42,43]. Supporting this, our electrochemical analyses demonstrated that compounds with NO_2_ at position 6 exhibit the lowest reduction potentials in the series, consistent with a more accessible LUMO and thus greater ability to accept electrons. This property, essential for redox activation, correlates well with the higher biological activity observed [44].

It is also worth highlighting that the intentionally limited structural design—without modifications to the benzyl group or the N^2^ substituent—allowed the isolated effect of nitro group position to be clearly observed, providing valuable insight for future structure-based design. The findings suggest that subsequent optimization efforts should consider introducing either electron-donating or electron-withdrawing groups on the benzyl chain to modulate the inductive or resonance effects of the 6-nitro group.

Although none of the tested compounds matched the potency of NFX (IC_50_ = 21.5 μM), the selectivity exhibited by the most active derivatives—combined with their favorable electrochemical profiles and spectroscopic evidence of radical intermediates—supports their consideration as viable starting points for further structural optimization, aimed at improving their pharmacological profile and trypanocidal potency.

### 2.4. Chemistry

#### 2.4.1. Electrochemical Characterization

The electrochemical behavior of a series of N-substituted nitroindazolin-3-ones was evaluated by cyclic voltammetry (CV) using a hanging mercury drop electrode (HMDE) in DMSO. The aim was to characterize their redox profiles and explore structure–activity relationships relevant to their trypanocidal potential. Previous studies by our group have suggested that the biological activity of nitroaromatic compounds may involve enzymatic bioactivation by type-I nitroreductases, leading to the generation of reactive oxygen species (ROS) and subsequent oxidative stress [45].

**Figure 6:**
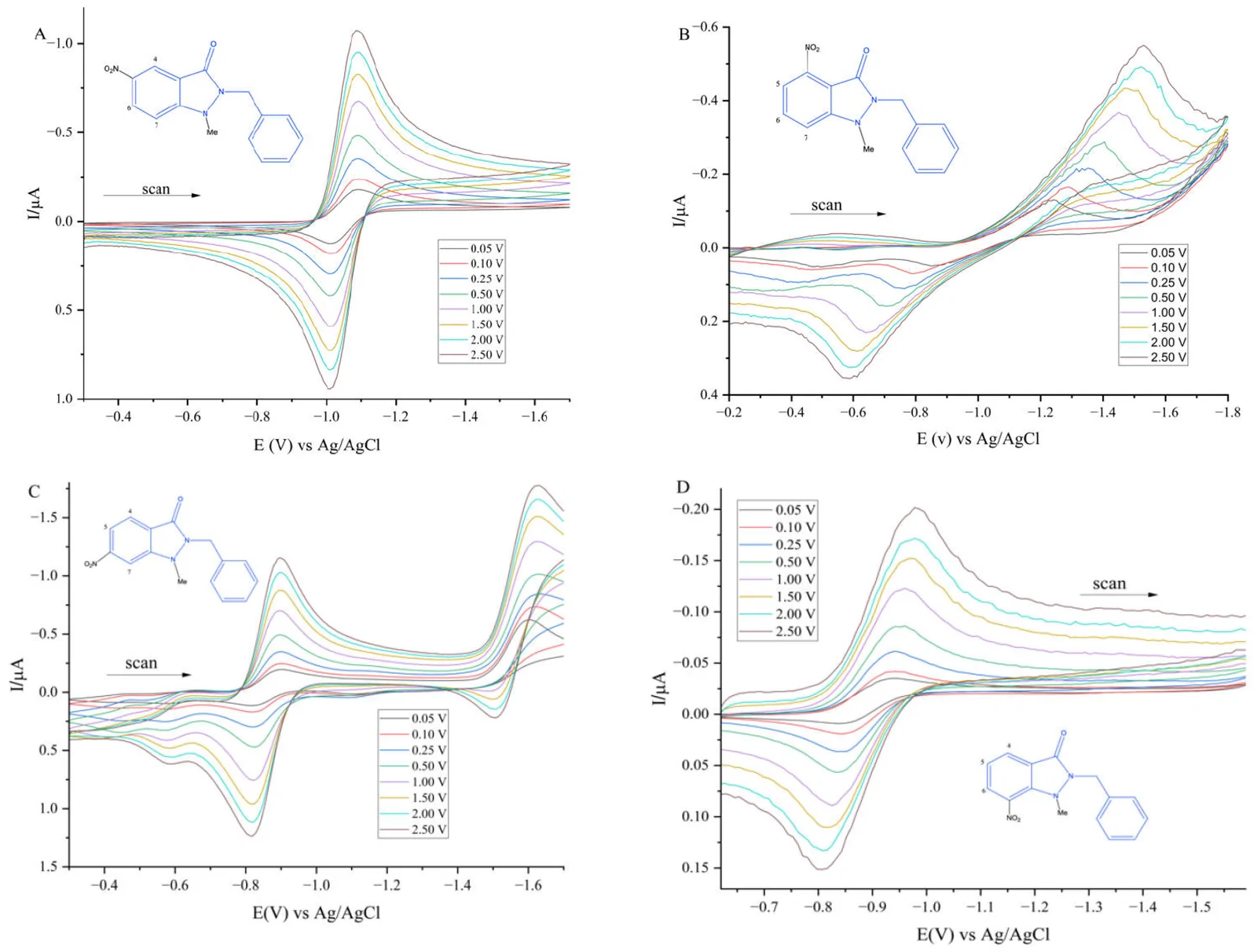
Representative cyclic voltammograms of 1,2-disubstituted nitroindazolin-3-ones derivatives recorded in DMSO using a hanging mercury drop electrode (HMDE), at scan rates ranging from 0.05 to 2.50 V/s. (**A**) Compound **1a** (NO_2_ at position 5). (**B**) Compound **1b** (NO_2_ at position 4). (**C**) Compound **1c** (NO_2_ at position 6). (**D**) Compound **1d** (NO_2_ at position 7).

Figure 6A shows a representative voltammogram for compound **1a**, bearing the nitro group at position 5, which displays a quasi-reversible redox couple, with a reduction peak at approximately −1.00 V and a reoxidation peak near −0.90 V vs. Ag/AgCl. This behavior is consistent with a one-electron reduction in the nitro group to a nitro radical anion, followed by partial reoxidation. Such patterns are typical of nitroheterocycles and have been reported in the literature [19]. A similar voltametric profile was observed for compounds **1d** and **2d**, where the nitro group is at position 7, suggesting comparable electronic and topological effects influencing the redox response.

Compounds **1b** and **2b**, both with the nitro group at position 4, exhibited distinct electrochemical behaviors. Figure 6B shows the voltammogram of compound **1b**, which displays a more negative reduction potential (−1.50 V) and an oxidation peak around −0.55 V. This suggests a less favorable reduction, possibly due to electronic destabilization caused by the adjacent carbonyl group (ortho to the nitro), which may lower the electron density at the nitro group. The anodic peak may correspond to the oxidation of a hydroxylamine intermediate, indicating a two-electron reduction pathway without accumulation of the nitro radical anion.

Conversely, compounds **1c** and **2c**, with the nitro group at position 6, exhibited the most complex electrochemical behavior. Figure 6C shows the voltammogram for **1c**, where two distinct cathodic peaks are visible (−0.90 V and −1.60 V), suggesting a sequential reduction process: formation of a nitro radical anion followed by reduction to a hydroxylamine. On the reverse scan, multiple anodic peaks appear—a reversible wave at −0.82 V (reoxidation of the radical), a peak at −1.50 V (hydroxylamine oxidation), and another at −0.56 V, likely corresponding to a nitroso intermediate. This rich redox chemistry may be favored by greater lowest unoccupied molecular orbital (LUMO) delocalization toward the nitro group at position 6, facilitating multi-electron transfer events [44].

Finally, compounds **1d** and **2d**, featuring a nitro group at position 7, exhibited voltammograms similar to that of **1a**, as illustrated in Figure 6D for compound **1d**. A single quasi-reversible couple is observed, consistent with a controlled one-electron reduction process. The similarity with compounds, having the nitro at position 5, suggests that both positions provide an electronic environment compatible with a stable radical anion and limited follow-up reactions.

Table S1 summarizes the half-wave reduction potentials (E_1_/_2_) of all compounds. Notably, b-type derivatives (NO_2_ at position 4) exhibit the most negative potentials, indicating higher resistance to reduction (for more details, see Supplementary Materials). In contrast, the lowest potentials were observed for c-type compounds (NO_2_ at position 6), supporting the idea of enhanced LUMO availability and reactivity at that position. Regarding the nature of the N-substituent (Me vs. Pr), no significant differences in redox potentials were observed, indicating that the position of the nitro group is the main determinant.

To gain deeper insight into the electrochemical reduction mechanism and the electronic distribution of the 1,2-disubstituted nitroindazolin-3-ones, we carried out an in situ spectroelectrochemical electron paramagnetic resonance (EPR) study. The compounds were reduced at potentials previously determined by cyclic voltammetry, and their radical intermediates were monitored via EPR spectroscopy.

For all tested compounds, EPR signals were detected upon reduction, confirming the formation of radical species during the redox process. In all cases, the unpaired electron was found to be delocalized over the aromatic system, as evidenced by characteristic hyperfine splitting patterns.

**Figure 7:**
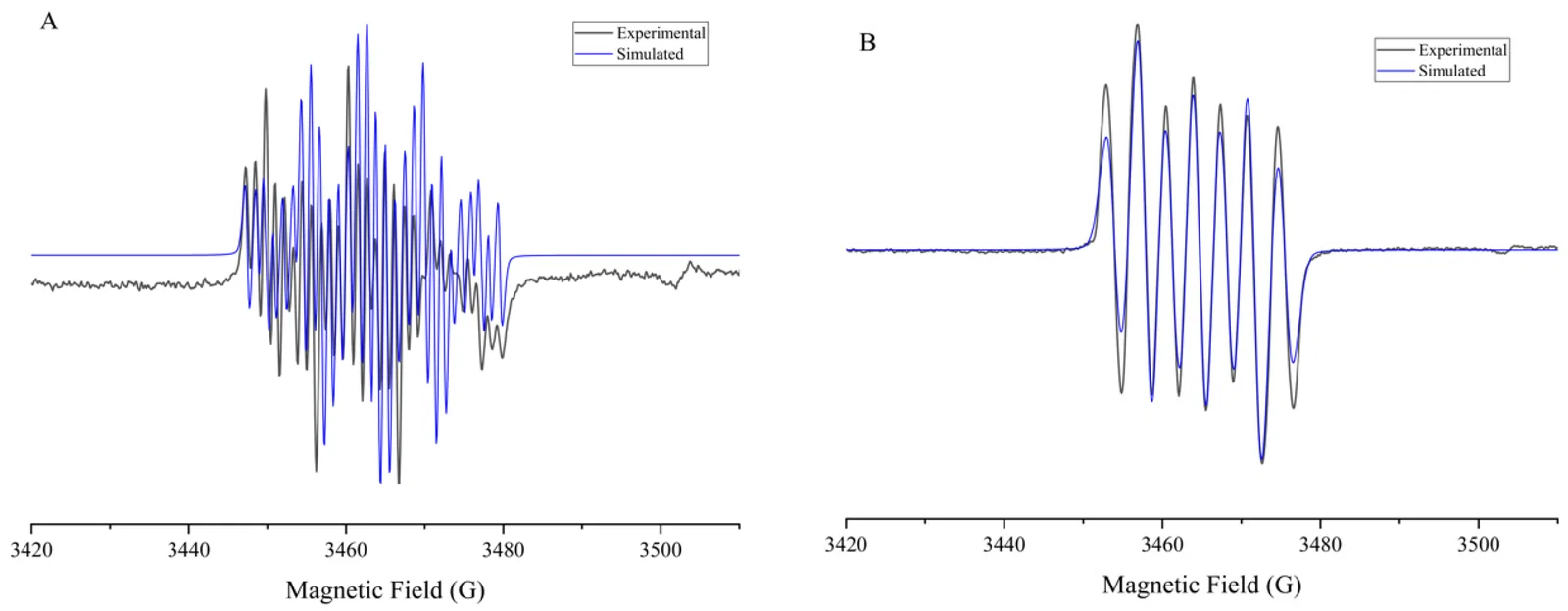

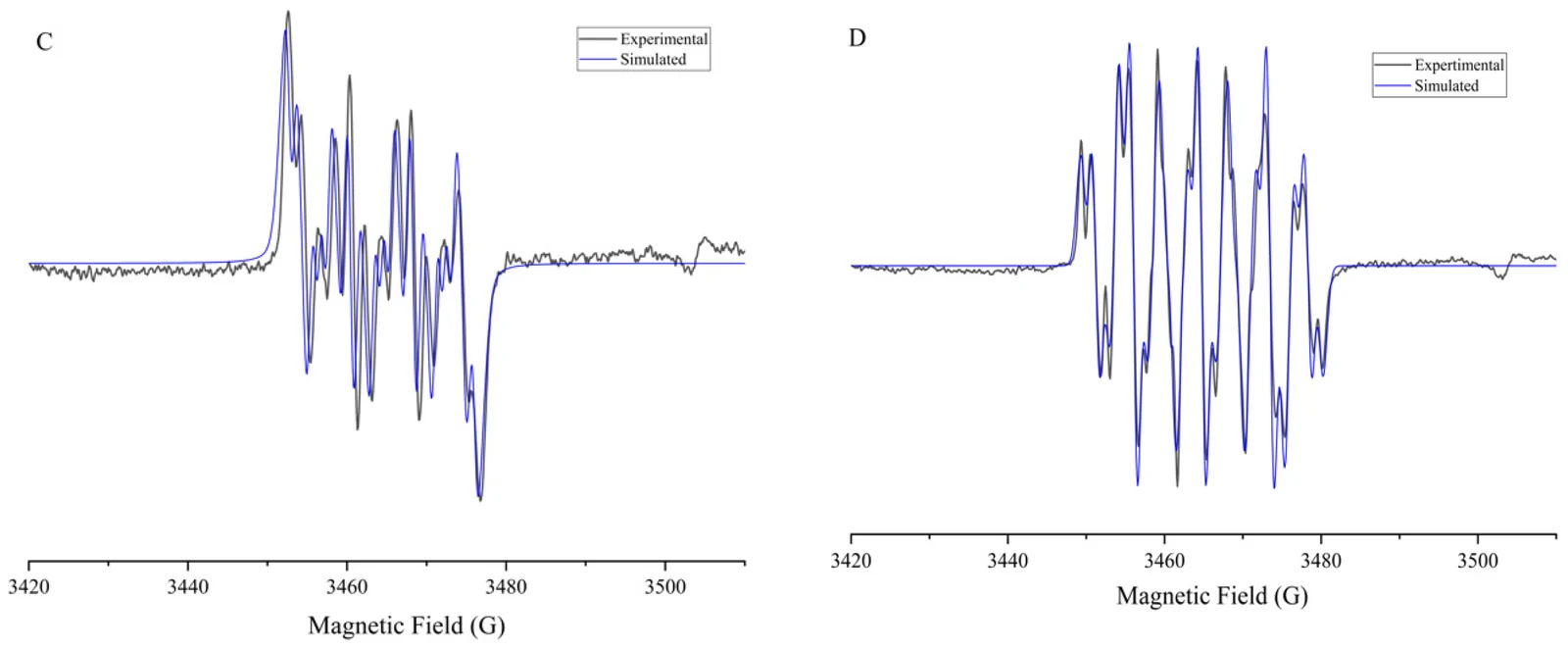
Experimental EPR spectra (black line) and fitted simulations (blue line) obtained after in situ electrochemical reduction in the selected compounds under anaerobic conditions. The spectra correspond to: (**A**) compound **1a** (nitro group at position 5), (**B**) compound **1b** (position 4), (**C**) compound **1c** (position 6), and (**D**) compound **1d** (position 7). Experiments were performed in DMSO at room temperature using rapid trapping in an electrochemical cell.

Notably, only compounds **1a** and **2a**, bearing the nitro group at position 5, exhibited an EPR pattern consistent with additional coupling to the N_2_ nitrogen of the fused pyrazole ring. In particular, the spectrum shown in Figure 7A displays 29 distinct lines, consistent with the superposition of two triplets (from the NO_2_ group and N^2^ nitrogen) and three doublets corresponding to aromatic protons (H_4_, H_6_, and H_7_). This suggests that the radical anion is not only delocalized over the nitro group and benzene ring but also extends into the fused heterocycle, indicating a broader electronic delocalization.

The fitted hyperfine coupling constants for compounds **1a** and **2a** (aNO_2_ > 10 G; aN^1^ ≈ 1.4 G) support this interpretation and point toward an electron-donating character of the substituent at N_2_, which appears to prevent spin migration into the aromatic side chain—an effect previously discussed in related systems [19].

In contrast, compounds bearing the nitro group at positions 4, 6, and 7 (**1b**–**1d**) showed no detectable coupling with the N^1^ nitrogen and exhibited lower hyperfine coupling constants with the NO_2_ group (6.8–8.8 G), suggesting a more localized spin density on the aromatic ring and reduced participation of the nitro moiety in radical stabilization.

Additionally, Figure 7B–D illustrate the EPR spectra of compounds **1b** (with NO_2_ at position 4), **1c** (at position 6), and **1d** (at position 7), respectively. These spectra exhibit simpler hyperfine patterns, consistent with restricted delocalization of the unpaired electron and exclusive localization within the aromatic framework. The lower complexity of these spectra correlates well with their electrochemical behavior, as previously described. Compound **2b**, which bears the most negative reduction potential, also exhibits the least stabilized radical anion, as evidenced by narrow coupling values and minimal spin delocalization. Compounds **2c** and **1d** display slightly broader patterns, and higher couplings for H_6_ and H_7_, suggesting a modest increase in conjugation and radical stabilization, particularly in **1d**, where the NO_2_ group in position 7 may interact more effectively with the π-system.

Together, these findings establish a clear relationship between the position of the nitro group, the electrochemical reducibility, and the topology of the radical anion formed during the reduction process. Only the 5-substituted derivatives exhibit extended delocalization involving the fused heterocycle. The complete set of simulated hyperfine coupling constants is summarized in Table S2.

In addition to the differences attributed to the nitro group’s position, we examined whether the nature of the substituent at N^1^ (methyl in series 1 vs. propyl in series 2) could influence the electronic distribution of the radical anion. Although both groups exert a mild electron-donating inductive effect (+I), the propyl group is bulkier and more flexible, potentially affecting the local geometry and conjugation within the indazolic framework.

In the simulated EPR spectra, no significant differences were observed in the number of lines or in the main hyperfine couplings, between analogous compounds from series 1 and 2. However, in derivatives bearing the NO_2_ group at position 5 (**1a** vs. **2a**), a slight decrease in coupling to the pyrazole nitrogen (N^1^) was observed, for the propyl-substituted compound (aN^1^ ≈ 1.35 G) compared to the methyl analog (aN^1^ ≈ 1.42 G). This may reflect a slightly reduced delocalization of spin density toward the fused heterocycle, possibly due to steric or torsional effects induced by the propyl group.

This effect was not evident in the compounds with the nitro group at other positions, where N^1^ does not participate significantly in spin delocalization. Thus, while the N^1^ substituent does not substantially alter the radical’s topology, it may exert a secondary influence on electronic delocalization, particularly in systems where the radical engages the heterocyclic core.

#### 2.4.2. Computational Analysis of Electronic Properties

To correlate the position of the nitro group with the electronic distribution and to gain insight into the electrochemical reduction process, DFT calculations were performed at the M06-2X/def2-TZVPP level for two series of N^1^-substituted 2-benzyl-1,2-dihydro-3*H*-indazol-3-one derivatives, each comprising four positional nitro isomers. The analysis encompassed frontier molecular orbital energies (highest occupied molecular orbital, HOMO, and LUMO), global reactivity descriptors (electron affinity, ionization potential, chemical hardness, and electrophilicity index), the nucleophilic Fukui index (***f*^+^**), and the spin density distribution of the radical anion.

**Figure 8:**
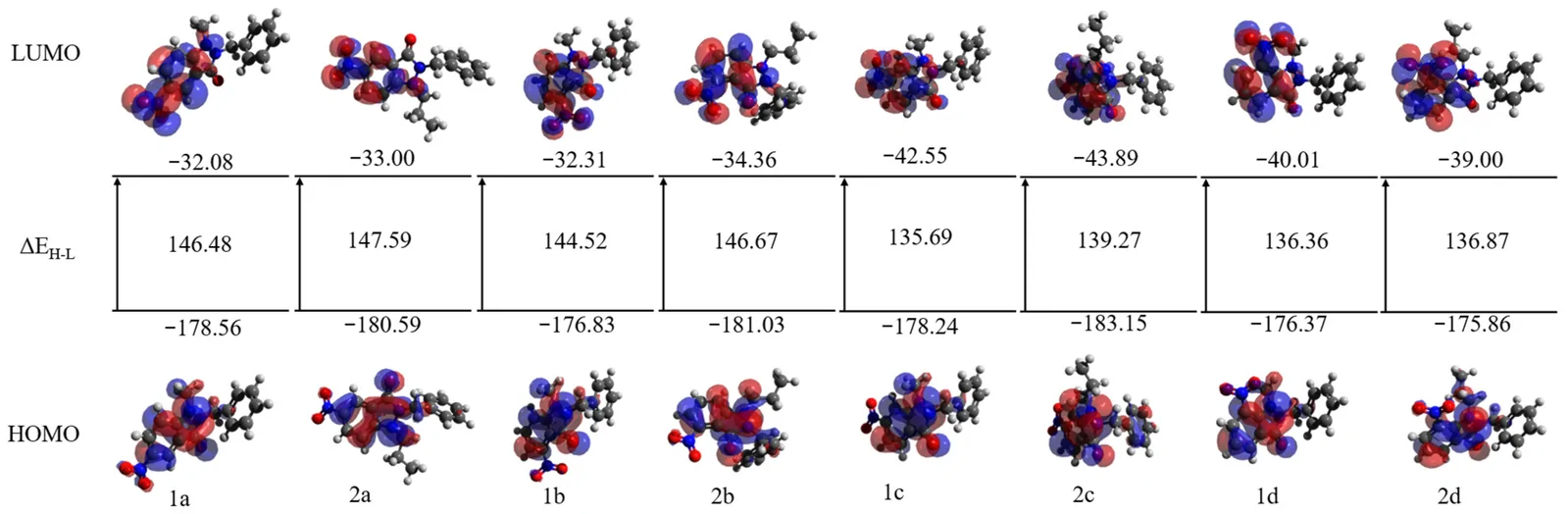
HOMO and LUMO frontier molecular orbitals in kcal/mol of compounds **1a**–**d** and **2a**–**d**.

As shown in Figure 8, compounds **1c** and **2c**, bearing the NO_2_ group at the C6 position, exhibited the most negative LUMO energies in the series (−42.55 and −43.89 kcal·mol^−1^, respectively), indicating a higher tendency to accept electrons. These values were accompanied by high vertical electron affinities (EAv = 25.23 and 27.02 kcal·mol^−1^) and the highest electrophilicity indices (ωv = 71.00 and 73.23 kcal·mol^−1^), suggesting a strong propensity to stabilize the negative charge formed during the reduction process. Additionally, these compounds showed some of the lowest chemical hardness values (ηv = 83.96 and 83.69 kcal·mol^−1^), which implies greater electronic flexibility to accommodate an incoming electron. Their ΔE_H-L_ values (135.69 and 139.27 kcal·mol^−1^) were also the lowest among all derivatives, reinforcing their highly polarizable character. Altogether, these parameters identify the C6-nitro derivatives as the most electronically favorable systems for efficient single-electron reduction and stable radical formation.

In contrast, compounds **1b** and **2b**, featuring the NO_2_ group at the C4 position, displayed significantly lower electron affinities (15.42 and 18.17 kcal·mol^−1^) and electrophilicity indices (ωv = 60.27 and 63.63 kcal·mol^−1^), along with a more localized LUMO distribution (Figure 8). This reduced electronic accessibility is consistent with their more negative experimental reduction potentials, as well as with the lower spin density observed in the nitro group, which suggests reduced efficiency in stabilizing the resulting radical species.

Global reactivity descriptors were evaluated by using two complementary approaches: the Koopmans’ theorem approximation (k), which estimates the electron affinity (EA) and ionization potential (IE) from the energies of the LUMO and HOMO orbitals, and the vertical approach (v), which calculates EA and IP from the total energies of the anion and cation, maintaining the geometry of the neutral species fixed. As detailed in Table S3, both methods yielded consistent trends; however, systematic differences were observed: EAv values were consistently lower than EAk values, reflecting the tendency of the LUMO to overestimate anion stability, whereas IEv values were higher than IEk values as expected, since vertical electron removal involves overcoming a higher energy barrier than predicted by the HOMO energy. This difference was particularly evident for compound 2c, where the EA calculated via Koopmans (EAk = 43.89 kcal·mol^−1^) overestimated the electron-accepting ability, relative to the more realistic vertical value (EAv = 27.02 kcal·mol^−1^). These findings underscore the need to consider the effects of electronic reorganization, in redox mechanism studies, and support the vertical approach as a more accurate descriptor of electronic reactivity in these systems.

**Figure 9:**
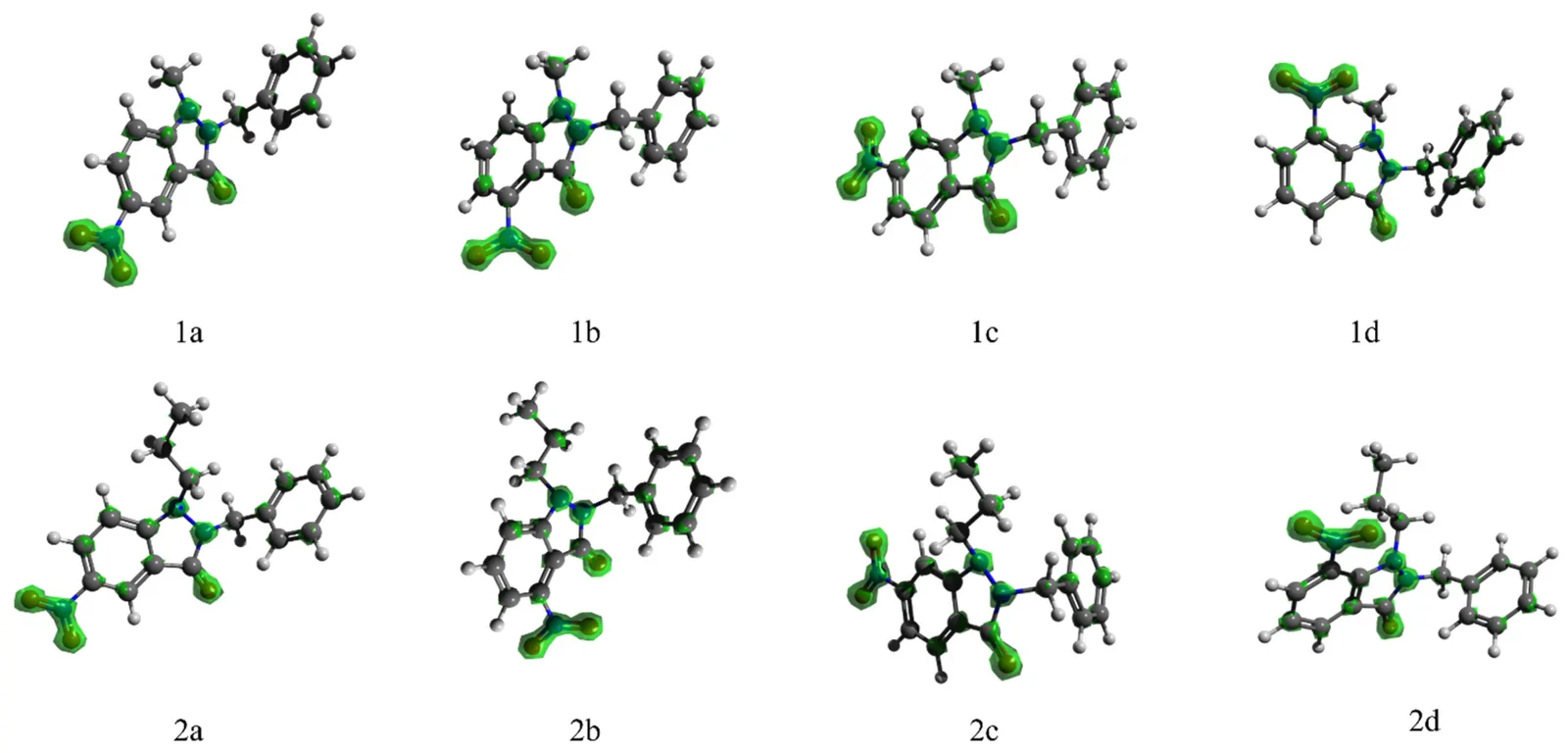
Isosurfaces of the nucleophilic Fukui (***f*^+^**).

**Figure 10:**
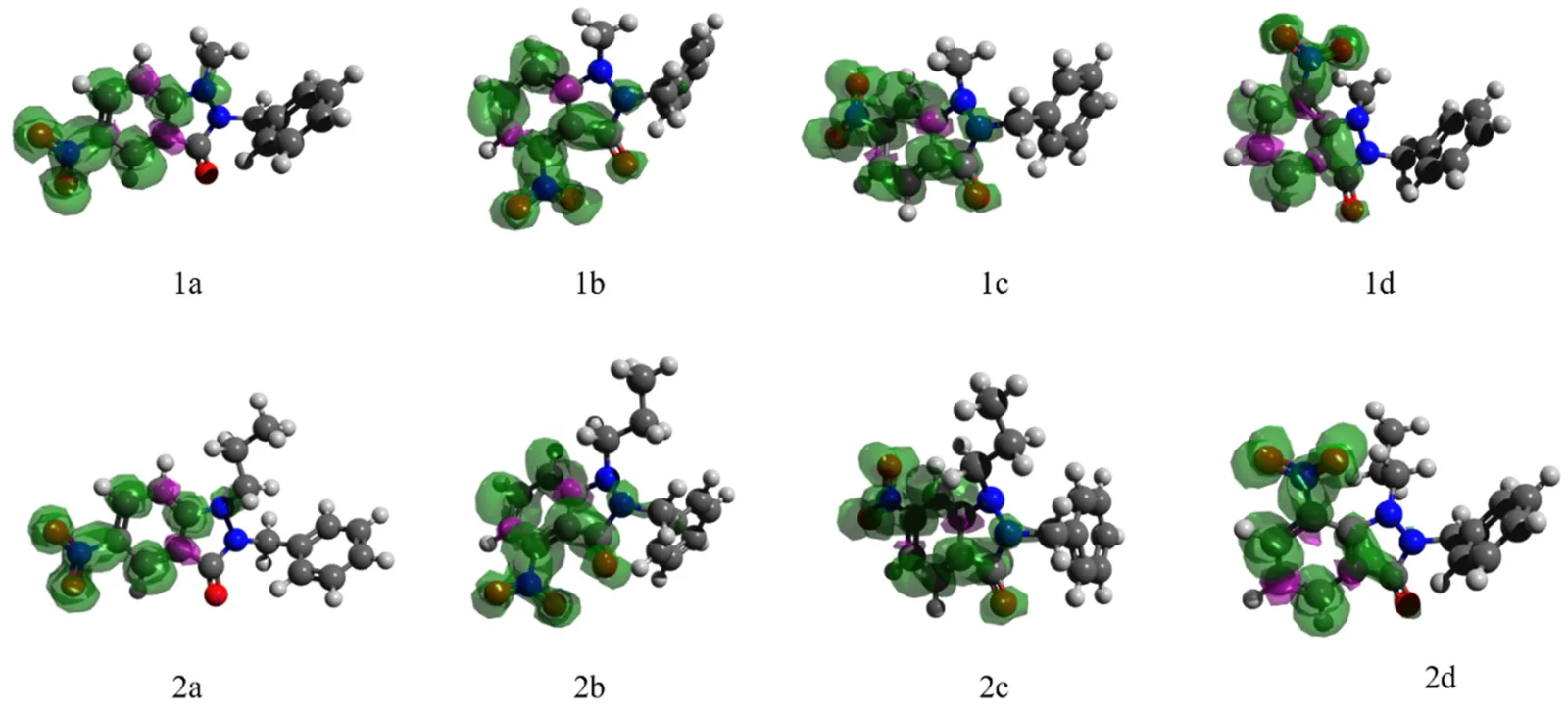
Spin densities $\rho_{spin}$ (RA) of the radical anion isosurfaces: in green the α spin density and in purple the β spin density.

The analysis of the ***f*^+^** and the spin density of the radical anion, illustrated in Figure 9 and Figure 10, provided insights into the molecular regions most susceptible to nucleophilic attack—an essential step in the reduction mechanism. In all compounds, the highest ***f*^+^** and *ρ*_spin_(RA) values were located on the atoms of the nitro group (atoms 18, 19, and 20). Notably, compounds **1a** and **2a** (NO_2_ at C5) showed a broader delocalization of the ***f*^+^** index over the indazole ring, particularly toward the nitrogen and carbon atoms adjacent to the fused pyrazole moiety (atoms 11 and 12). This pattern aligns with simulated EPR results and suggests that, in these compounds, the electronic distribution favors the delocalization of the unpaired electron, contributing to radical stabilization.

By contrast, in compounds **1c** and **2c**, the ***f*^+^** index was strongly localized over the nitro group and was accompanied by high spin density (*ρ*_spin_(RA) > 0.13) on the same atoms. This configuration suggests that the added electron is incorporated in a more localized yet highly efficient manner, facilitating direct electron transfer and the effective stabilization of the radical anion. This behavior is consistent with their high ω values and low chemical hardness. Conversely, compounds **1b** and **2b** exhibited the lowest ***f*^+^** and *ρ*_spin_(RA) values in the nitro region, correlating with their poor electrochemical performance and confirming their less favorable redox profiles (Table S4).

Taken together, the computational results demonstrate that the position of the nitro group plays a decisive role in shaping the electronic profile and redox reactivity of these systems. C6-nitro derivatives (**1c** and **2c**) emerged as the most suitable candidates for single-electron reduction processes, owing to their high electron-accepting ability, elevated electrophilicity, and localized spin density. The effective conjugation of the NO_2_ group with the aromatic system and its electronic alignment with the carbonyl moiety contribute to the stabilization of the resulting radical anion, a key factor for enzymatic redox bioactivation processes, including those previously reported for *T. cruzi* nitroreductases. The integration of Koopmans and vertical approaches not only validates the observed trends but also highlights the importance of accounting for electronic reorganization in redox studies. Ultimately, the combined analysis of LUMOs, Fukui indices, and spin densities offers a robust framework for predicting and rationalizing the redox behavior of nitroaromatic compounds, with therapeutic potential.

**Scheme 1:**
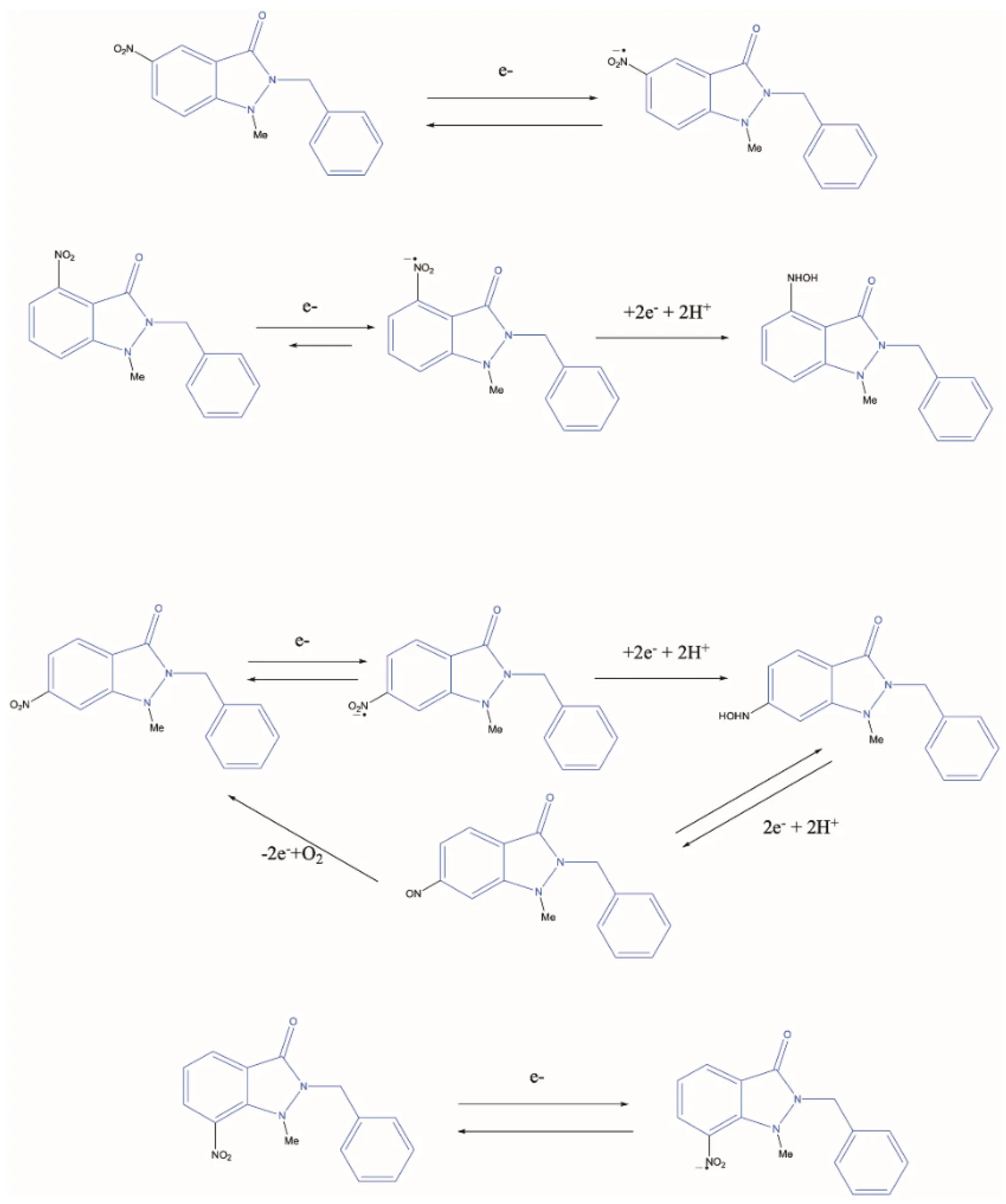
Proposed electrochemical reduction mechanism for the nitroindazolinone derivatives of **a** series. The sequence involves the stepwise formation of a nitro radical anion, followed by reduction to the nitroso and hydroxylamine species, and partial reoxidation to the nitroso intermediate under electrochemical conditions. No full regeneration of the nitro group was observed under the experimental conditions.

Finally, Scheme 1 integrates the findings obtained from cyclic voltammetry, EPR spectroscopy, and DFT calculations into a coherent electrochemical reduction mechanism proposed for the compounds of the **a** series. The mechanism involves the initial formation of a nitro radical anion (Ar–NO_2_•^−^), followed by stepwise reduction to the nitroso species (Ar–NO) and subsequent conversion to the corresponding hydroxylamine (Ar–NHOH), through a concerted two-electron, two-proton transfer process. Electrochemical reoxidation allows for partial recovery of the nitroso intermediate, thus establishing a redox cycle that accounts for both the voltametric behavior and the theoretically predicted electronic distribution.

### 2.5. Oxidative Stress and Spin Trapping Analysis by ESR

To further investigate the potential mechanism of action underlying the differential biological effects observed between the 5-nitro and 6-nitro substituted compounds, an electron spin resonance spin trapping study was conducted by using DMPO (5,5-dimethyl-1-pyrroline-N-oxide) as the spin trap. This technique enables the indirect detection of short-lived radical intermediates, generated under physiological-like conditions, and has been extensively validated for the identification of reactive oxygen species (ROS), particularly hydroxyl radicals (•OH).

The experiments were performed under aerobic conditions in the presence of NADPH, simulating a reducing intracellular environment. Upon incubation with selected compounds from both the 5-nitro and 6-nitro series, characteristic ESR signals of DMPO adducts were detected. In all cases, the formation of DMPOX (5,5-dimethyl-2-oxopyrrolidine-1-oxyl), an oxidized paramagnetic species derived from DMPO, was observed. This species is widely accepted as a marker of oxidative stress, resulting from redox cycling of nitroaromatic compounds in the presence of oxygen and NADPH [46].

In addition to DMPOX, a second species was identified as the hydroxyl radical adduct (DMPO–OH), displaying a diagnostic hyperfine splitting pattern (aNN ≈ aNH ≈ 14.00 G), and marked in the spectrum with a “+” symbol. The detection of •OH is particularly significant, as this radical is one of the most reactive and cytotoxic ROS, and its formation has been directly associated with oxidative-stress-mediated apoptosis in *T. cruzi* and other intracellular pathogens.

Interestingly, no other spin adducts were detected in any of the compounds analyzed, suggesting that the redox activation process proceeds predominantly toward hydroxyl radical formation, rather than producing alternative intermediates such as superoxide or organic peroxides. This selectivity may be associated with mono-electronic nitroreduction processes involving enzymatic redox activation of the nitro group, followed by oxygen-mediated radical propagation, as previously described for nitroaromatic compounds in *T. cruzi* [19,44].

While no qualitative differences were observed in the type of radical species generated between the 5-nitro and 6-nitro compounds, a notably higher signal intensity was detected in the 6-nitro derivatives, especially at intermediate incubation times. This increase in signal may reflect more efficient or sustained ROS generation, which aligns with the greater trypanocidal activity exhibited by these compounds and supports the hypothesis that their biological effect is at least partially mediated by intracellular oxidative stress. Although radical-mediated oxidative stress appears to play a central role, alternative or complementary bioactivation pathways described for nitroheterocycles cannot be excluded.

**Figure 11:**
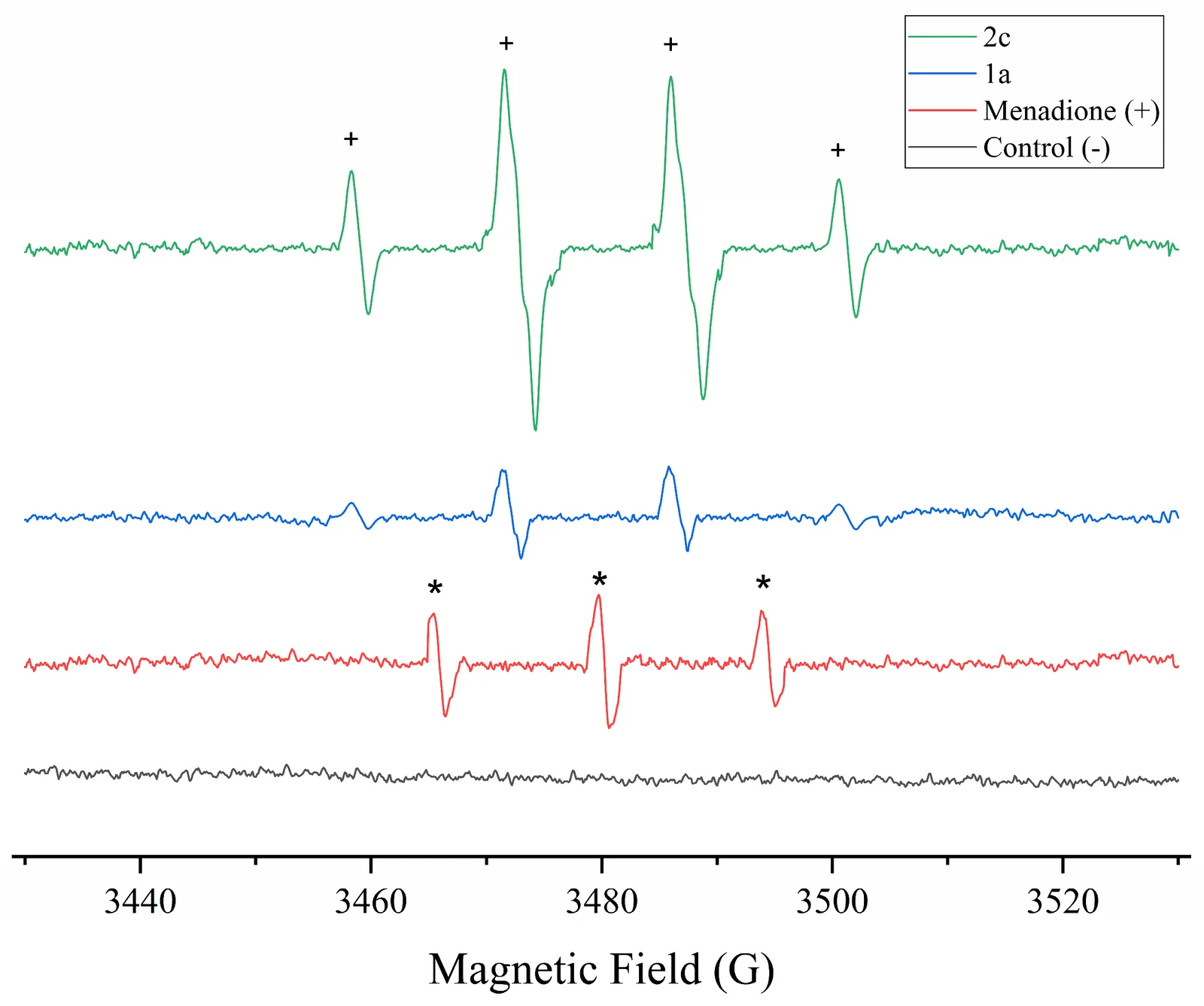
EPR spectra obtained after incubation of selected compounds with DMPO (spin trap) in the presence of NADPH under aerobic conditions. Signals are shown for compound 2c (green), **1a** (blue), positive control (menadione, red), and negative control (no compound, black). The spectrum of compound **2c** displays characteristic signals of the DMPO–OH adduct, attributable to hydroxyl radical (•OH) formation, with hyperfine couplings of aN ≈ aH ≈ 14.0 G, marked with “+”. Menadione predominantly generates the oxidized DMPO species (DMPOX), marked with an asterisk (“*”). No additional radical species were detected. These results suggest that the evaluated compounds induce oxidative stress through the generation of intracellular hydroxyl radicals, which is part of their mechanism of action.

Collectively, these findings suggest that the mechanism of action of the most active compounds involves the generation of intracellular hydroxyl radicals as a result of enzymatic redox activation, leading to apoptosis in *T. cruzi*. Figure 11 illustrates the representative signal of the DMPOX adduct and the characteristic peak corresponding to hydroxyl radical formation (marked with “+”) obtained under aerobic conditions in the presence of both NADPH and DMPO.

## 3. Materials and Methods

### 3.1. Computational Studies

#### 3.1.1. Dataset and Design of Training and Prediction Sets

We used a freely available dataset from the Drugs for Neglected Diseases initiative (DNDi), downloaded from the ChEMBL site (https://chembl.gitbook.io/chembl-ntd, accessed on 10 August 2025, Deposited Set 14). It is composed of 583 compounds experimentally assayed against *T. cruzi* and is of great structural diversity. Compounds with values of IC_50_ ≤ 50 μM (322 compounds) were considered as active ones, and compounds with greater values were considered as inactive ones (261 compounds).

For the purpose of designing the training and test set (sometimes known as prediction set), as well as to assess the structural variability between these series, two types of cluster analyses (CAs) were performed for both groups of compounds (active and inactive ones) by using the STATISTICA Version 6 software [47]. This software has two types of cluster analysis, namely hierarchical nearest-neighbor cluster analysis (k-NNCA) and k-means cluster analysis (k-MCA). The number of members in every cluster, and the standard deviation of the variables in the cluster (kept as low as possible), were considered to have an acceptable statistical quality of data partition into clusters. The values of the standard deviation (SD) between and within clusters, of the respective Fisher ratio, and their p-level of significance were also examined.

First, we proceeded to standardize all previously calculated molecular descriptor matrices. Then, a hierarchical cluster analysis was performed to visualize the compounds distribution in different groups, and to detect repeated compounds or outliers in the database. Afterwards, a k-MCA was carried out to obtain, in a rational and representative way, both the training set (TS) and the prediction set (PS); then, we select randomly from each cluster about 25% of the compounds belong to the PS. Finally, the composition of the series was: (243/199) for a total of 442 compounds in TS, and (79/62) for a total of 141 in PS. The entire dataset with their identification codes, SMILES (simplified molecular-input line-entry system), experimental values, and their belonging to either TS or PS can be found in the Supplementary Materials.

#### 3.1.2. Molecular Descriptors

The 0-2D molecular descriptors were calculated with alvaDesc v3.0.14 (2026) software [48]. A total of 902 descriptors were used after elimination of constant, near-constant or correlated-at-85% variables. This software contains constitutional indices (0D); charge descriptors, atom-centered fragments, functional group counts, and molecular properties (1D); 2D autocorrelations, 2D matrix-based descriptors, burden eigenvalues, topological descriptors, information indices, walk and path counts, connectivity indices, edge-adjacency indices, and 2D atom pairs (2D).

#### 3.1.3. Classification Technique

Random Forest (RF) is an extension of classification and regression trees (CART). They perform well even in the presence of a large number of features and a small number of observations [49,50]. In RF, each tree is assembled by using a different bootstrap sample; each node is split by using the best features among a subset of predictors randomly chosen at that node. This process is fast even for big datasets with successful results, and the variable importance and the reduction in the number of features can be measured. It is important to point out that although random forests perform well in many applications, their theoretical properties are not fully understood [51]. We used Python scripts developed by utilizing the Scikit-learn package to develop the RF model [52]. In the case of RF, two main parameters were varied. The number of trees (n_estimators) was changed from 1 to 200 in intervals of one. In addition, the max_depth of the forest was assessed from 1 to 20.

#### 3.1.4. Applicability Domain

“The applicability domain of a (Q)SAR model is the response and chemical structure space in which the model makes predictions with a given reliability” [53]. This means that any QSAR model must be related to a defined applicability domain (AD). This is derived from the fact that all QSARs inevitably present limitations associated with the properties and the chemical structures, as well as with mechanisms of action for which the models can make reliable predictions [54]; only the predictions for chemicals falling within this AD can be considered reliable and not model extrapolations [55]. The AD inside the chemical space is defined as the hypothetical area defined by model’s descriptors and modeled response, and thus by the compounds that belong to the training set, through the specific MDs present in the model. Specifically, the AD of a QSAR model is the range within which it tolerates a new molecule [56].

#### 3.1.5. Feature Importance Analysis

Understanding the biological significance of the selected descriptors can be challenging, as machine learning models are usually seen as “black boxes” due to their complex nature. The Shapley Additive Explanations (SHAP) method was used for model interpretation [57,58]. The values of SHAP, derived from coalitional game theory, were used to analyze the contribution of each feature to the model output. The calculation involves combining and weighting model outputs for different feature value combinations, to determine the marginal contribution of each feature value to the output. The importance of a feature *i* is given by its Shapley value (∅_i_), which is calculated as the average of its contributions across all possible combinations of a feature set.

### 3.2. Experimental Assays

#### 3.2.1. Chemical Assays

The synthesis and full characterization of the 4-, 6-, and 7-nitroindazolin-3-one derivatives corresponding to compounds **1b**–**d** and **2b**–**d** have recently been reported by Fonseca-Berzal et al. [34], including detailed synthetic procedures, analytical data, elemental analyses, and ^1^H and ^13^C NMR characterization [59,60]. The synthesis and characterization of the 5-nitro analogs **1a** and **2a** have also been previously reported [33]. In the present study, compounds **1a**–**d** and **2a**–**d** were used for computational, electrochemical, spectroscopic, and biological evaluation.

Cyclic Voltammetry (CV): Electrochemical studies were carried out on a Metrohm Autolab instrument model PGSTAT204 (Metrohm Autolab B.V., Utrecht, The Netherlands) by using dimethyl sulfoxide (DMSO) as the solvent (1 mM solutions), tetrabutylammonium perchlorate (0.1 M TBAP) as the supporting electrolyte, a hanging drop mercury electrode (HDME) as the working electrode, Ag/AgCl (3M KCl) as the reference electrode, and a graphite rod as the counter electrode. All measurements were performed after bubbling with nitrogen (N_2_) for 10 min. The studies of Electron spin resonance (ESR) tests were conducted with a Bruker ECS 106 X-band (9.85 GHz) spectrometer (Bruker BioSpin GmbH, Rheinstetten, Germany), with a rectangular cavity and a field modulation of 50 kHz. In situ reduction in the compounds under study was carried out at potentials determined by CV to generate radical species. The DMSO was used as the solvent, TBAP as the supporting electrolyte, and a platinum wire as the working electrode.

#### 3.2.2. Biological Studies

The trypomastigotes of the Dm28 strain of *T. cruzi* and mammalian cells were obtained from an in-house collection (Programa de Biología Integrativa, Facultad de Medicina, Universidad de Chile).

Cell cultures: VERO cells (ATCC CCL-81) were cultured in Roswell Park Memorial Institute 1640 medium (RPMI 1640), supplemented with 5% heat-inactivated fetal bovine serum (FBSi) and 1% antibiotics (penicillin-streptomycin). In this, and all cultures described below, cells were incubated at 37 °C, with 5% CO_2_ and 98% relative humidity. Cells at semi-confluence were harvested by trypsinization and sedimented by centrifugation at 500× *g* for 5 min at room temperature, re-suspended in RPMI 1640 medium, and counted with trypan blue by using the JuLI™ FL Live Cell Analyzer device (NanoEntek Inc., Seoul, Republic of Korea) [61]. The EA.hy926 cells (ATCC^®^ CRL-2922) were cultured in DMEM high glucose (Dulbecco’s Modified Eagle Medium) supplemented with 10% FBSi and 1% antibiotics (penicillin-streptomycin). In this and all cultures described below, cells were incubated at 37 °C with 5% CO_2_ and 98% relative humidity.

Parasite culture and harvesting *Trypanosoma cruzi*: VERO cells at semi-confluence were infected with trypomastigotes from the Dm28 strain and cultured in RPMI 1640 medium supplemented with 5% FBSi and 1% antibiotics (penicillin-streptomycin), at 37 °C in a humidified atmosphere with 5% CO_2_. The parasites invaded the cells and replicated intracellularly as amastigotes. After 48 to 72 h, amastigotes were allowed to differentiate back into trypomastigotes and were released by cell lysis. Trypomastigotes were separated from cellular debris by centrifugation at 500× *g* for 5 min and then recovered from the supernatant by centrifugation at 3500× *g* for 10 min at 4 °C, re-suspended in RPMI 1640 medium supplemented with 5% fetal bovine serum (FBS) and 1% antibiotics, and quantified by using a Neubauer chamber [62].

Cytotoxicity on cells: The EA.hy926 cells were seeded in 96-well plates, with 5 × 10^4^ cells per well, and incubated with the compounds (previously dissolved in DMSO), at concentrations ranging from 17 μM to 400 μM for 24 h at 37 °C under a humid atmosphere with 5% CO_2_. Triton X-100 was used as positive control, and untreated cells served as a negative control. Additionally, the maximum concentration of DMSO was used as a control for the method. After the incubation period, cells were washed with PBS, and 100 μL of DMEM high glucose with resazurin to a final concentration of 3 mM was added to each well, followed by incubation for 3 h at 37 °C under a humidified atmosphere with 5% CO_2_. After incubation, measurements were taken by using a microplate reader Thermo Scientific™ Varioskan^®^ Flash (Thermo Fisher Scientific Oy, Vantaa, Finland) at excitation wavelengths of 560 nm and emission wavelengths of 590 nm. The obtained values were expressed as IC_50_ [63].

Activity on *T. cruzi* trypomastigotes: Parasites at 10^7^ parasites/mL concentration were incubated for 24 h, with the compounds under investigation (previously dissolved in DMSO) at a concentration of 100 μM at 37 °C under a humid atmosphere with 5% CO_2_; DMSO concentration did not exceed 0.5% *v*/*v*. The NFX was used as a positive control for *T. cruzi*. Untreated parasites served as a negative control. Additionally, considering that the compounds were dissolved in DMSO, a control condition with the maximum possible DMSO concentration was used, to verify that this solvent alone has neither antiparasitic nor cytotoxic activity. Subsequently, parasites with their respective conditions were transferred to a 96-well white fluorescence reading plate, adding resazurin at a final concentration of 3 mM with 0.8 mM phenazine. The plate was incubated for 3 h at 37 °C under a humidified atmosphere with 5% CO_2_, and fluorescence measurements were taken by using a microplate reader (Thermo Scientific™ Varioskan Flash), at excitation wavelengths of 560 nm and emission wavelengths of 590 nm. Once the most active compound against the parasites was identified, the IC_50_ value, corresponding to the inhibitory concentration of 50 percent of the parasite population, was determined [64,65].

Subsequently, the most active compounds were added at the IC_50_ concentration, and fluorescence (excitation: 488 nm, emission: 528 nm) was recorded for 40 min by using a Thermo Scientific Varioskan Lux microplate reader. Over time, the area under the fluorescence increase curve was determined by using Origin 8 software (version 9.2), with normalization relative to the control. The results correspond to three independent experiments’ mean ± standard deviation (SD).

Electron Spin Resonance (ESR) or Electron Paramagnetic Resonance (EPR) Studies in Parasite Media: The free radical production capacity of the most active 6-nitrocoumarin-3-thiosemicarbazone hybrids, against each parasite, was evaluated by using electron spin resonance (ESR) spectroscopy, with 5,5-dimethyl-1-pyrroline-N-oxide (DMPO) as a spin-trapping agent [19,66].

Each tested hybrid was dissolved in spectroscopy-grade DMSO, at an approximate concentration of 1 mM, and added to a mixture containing *Trypanosoma cruzi* (Dm28c strain) at a density of 50 × 10^6^ trypomastigotes per mL, along with DMPO at a final concentration of 250 mM. Menadione (20 μM) was used as a positive control.

The mixture was transferred to a 50 μL capillary, and ESR spectra were recorded in the X-band (9.85 GHz) by using a Bruker ECS 106 spectrometer (Bruker BioSpin GmbH, Rheinstetten, Germany) with a rectangular cavity and 50 kHz field modulation. All spectra were acquired on the same scale after 15 scans.

### 3.3. Computational Details

All quantum chemical calculations were carried out by using the ORCA 6.0.1 software package [67]. Geometry optimizations of the neutral species were performed in the gas phase by using the density functional theory (DFT) M06-2X hybrid meta-GGA functional in combination with the def2-TZVPP basis set, without imposing symmetrical constraints. The M06-2X functional includes 54% Hartree–Fock exchange, and is parameterized for accurate treatment of noncovalent interactions and thermochemistry in organic molecules [68]. Vibrational frequency calculations were conducted at the same level of theory to confirm that all optimized structures correspond to true local minimum, as verified by the absence of imaginary frequencies [68,69].

Electronic properties were computed by using two complementary approaches: Koopmans’ theorem and the more correct vertical method. In the vertical method, the single-point energies of the corresponding cationic (E_N−1_) and anionic (E_N+1_) species were calculated by using the optimized geometry of the neutral molecule (EN), where N is the number of electrons in the neutral system [68,70,71].

Ionization Energy (IE) = (E_N−1_) − (E_N_),

Electron Affinity (EA) = (E_N_) − (E_N+1_)

Chemical Potential (μ) represents the escaping tendency of electrons from a system:

$$\mu =\frac{\left(IE+EA\right)}{2},$$

Chemical Hardness (η) reflects the resistance to charge transfer:

$$\eta =\frac{\left(IE-EA\right)}{2},$$

Global Electrophilicity Index (ω) estimates the electrophilic character of the system:

$$\omega =\frac{\mu^{2}}{2\eta }$$

To evaluate local reactivity, two atomic-level descriptors were computed:

Condensed nucleophilic Fukui function $f_{k}^{+}$ identifies atoms in the molecule that are more susceptible to electrophilic attack. It was calculated by using the finite difference approximation as:

$$f_{k}^{+}=qk\left(N+1\right)-qk(N)$$

where qk(N) and $qk\left(N+1\right)\;$ are the Hirshfeld charges of atom k in the neutral and anionic species, respectively [72,73].

Condensed spin density $\rho_{k}^{spin}$ is defined as the difference between alpha and beta electron densities on each atom *k* in the radical anion:

$$\rho_{k}^{spin}=\rho_{k}^{\alpha }\;-\;\rho_{k}^{\beta }$$

This descriptor provides insight into the delocalization of the unpaired electron and the radical stabilization potential [73].

All condensed values were obtained by using Hirshfeld population analysis, as implemented in ORCA [67]. Visualization of the Fukui function isosurfaces was carried out by using Multiwfn version 3.8 [74], and molecular structures, molecular orbitals, and spin density distributions were rendered by using Avogadro version 1.2.0. All calculations including those for neutral, anionic, and cationic species were consistently performed at the DFT M06-2X/def2-TZVPP level of theory, ensuring methodological coherence across descriptors and minimizing the influence of basis set and structural artifacts.

## 4. Conclusions

Herein we present a novel machine learning-based model developed by using alvaDesc molecular descriptors for the classification of chemical compounds as active or inactive against *Trypanosoma cruzi*. The resulting models enable *in silico* screening of both physical and virtual compounds, thereby supporting the rational discovery of new lead candidates for the chemotherapy of trypanosomiasis. This approach offers reliable predictions of antitrypanosomal activity, enhancing the efficiency of early-stage drug discovery while reducing resource consumption. By using the RF model developed here, a group of 1,2-disubstituted nitroindazolin-3-ones was evaluated, predicting their potential antitrypanosomal activity. The compounds were synthesized and subsequently evaluated *in vitro* against *T. cruzi*, to corroborate the predictive reliability of the machine learning classification model developed in this study. The results obtained from several chemical and biological experiments validate the reliability of the classification model developed here. Moreover, these results allow us to establish a clear relationship between chemical structure and biological activity, highlighting the influence of the nitro group position within the indazolin-3-one scaffold on the redox properties of the compounds, particularly their reduction potentials and propensity for radical formation. Based on the combined electrochemical, spectroscopic, computational, and biological evidence, a plausible mechanism of action can be proposed, in which redox activation of the nitro group may promote the generation of reactive oxygen species through enzymatic redox processes previously described in *T. cruzi*. For the most active compounds, the data are consistent with intracellular hydroxyl radical generation, contributing to oxidative-stress-mediated parasite death. Finally, we can say that the algorithm developed in this study represents a significant step forward, in the search for efficient strategies to discover new antitrypanosomal compounds. It exemplifies how rational, computer-aided approaches can streamline early-stage drug discovery by reducing associated costs and accelerating the progression of novel chemical entities through the development pipeline.

## Figures and Tables

**Table 1:** Prediction performances for the RF model for the training and test sets.

H-049, VE2sign_B(m), SsssCH, SpMin3_Bh(i), s3_numAroBonds, X1A				
	Accuracy	Sensibility	Specificity	*fpr* ^1^
Training set	0.821	0.798	0.849	0.151
Test set	0.787	0.734	0.855	0.145

^1^ *fpr*: false positive rate.

**Table 2:** Trypanocidal activity of 1,2-disubstituted nitroindazolin-3-ones derivatives against *T. cruzi*: prediction with RF model, trypomastigote form (Dm28c strain), cytotoxicity in VERO and EA.hy926 mammalian cell lines, and selectivity indices (SI) calculated as the ratio of IC_50_ in mammalian cells to IC_50_ in parasites. Values are expressed as mean ± SD from at least three independent experiments.

Compounds	Pred.	IC_50_/µM			SI *
		Trypomastigote of *T. cruzi* Dm28c	VERO Cells	EA.hy926 Cells	
**1a**	I	189.7 ± 1.4	>300	>300	>1.58
**2a**	I	149.6 ± 2.0	>300	>300	>2.00
**1b**	I	164.4 ± 1.3	>300	>300	>1.82
**2b**	I	170.6 ± 1.1	>300	>300	>1.76
**1c**	I	71.3 ± 0.9	>300	>300	>4.21
**2c**	A	40.9 ± 0.8	>300	>300	>7.33
**1d**	I	212.4 ± 1.3	>300	>300	>1.41
**2d**	I	142.1 ± 2.1	>300	>300	>2.11
**NFX**		21.5 ± 1.4	>300	>300	>13.95

* Selectivity Index IC_50_ mammalian cell/IC50 trypomastigote Dm28c.

## Data Availability

The original contributions presented in the study are included in the article/Supplementary Materials, further inquiries can be directed to the corresponding authors.

## Supplementary Materials

The following supporting information can be downloaded at: https://www.mdpi.com/article/10.3390/ph19091473/s1, Table S1: Cathodic (Ec) and anodic (Ea) peak potentials obtained by cyclic voltammetry for compounds **1a**–**2d**, recorded at a scan rate of 2.00 V/s in DMSO using a hanging mercury drop electrode (HMDE). The table also includes the calculated half-wave potentials (E_1/2_) for the observed redox couples; Table S2: Hyperfine coupling constants of 1,2-disubstituted nitroindazolin-3-ones obtained by semi-empirical computation; Table S3: Calculated global electronic properties (in kcal/mol) of the studied compounds. The table includes the energies of the highest occupied molecular orbital (HOMO, H) and the lowest unoccupied molecular orbital (LUMO, L), the HOMO–LUMO energy gap (Δε_H–L_), electron affinity (EA), ionization potential (IE), chemical hardness (η), and electrophilicity index (ω). k: Values calculated based on Koopmans’ theorem/v: values obtained using the vertical approach to evaluate the electronic properties, keeping the geometry of the neutral state fixed; Table S4: Nucleophilic Fukui index (*f*^+^) and spin densities $\rho_{spin}$(RA) of the radical anion for compounds **1a**–**1d** and **2a**–**2c**.

## Abbreviations

The following abbreviations are used in this manuscript:

Act	Activity
AD	applicability domain
AUC	Areas Under the Curve
BZN	benznidazole
CD	Chagas disease
CA	cluster analyses
CV	cyclic voltammetry
DNDi	Drugs for Neglected Diseases initiative
EPR	electron paramagnetic resonance
ESR	Electron Spin Resonance
HMDE	hanging mercury drop electrode
HOMO	highest occupied molecular orbital
IC_50_	half-maximal inhibitory concentration
k-NNCA	k nearest-neighbor cluster analysis
k-MCA	k-means cluster analysis
LUMO	lowest unoccupied molecular orbital
NFX	nifurtimox
PS	prediction set
QSARs	quantitative structure–activity relationships
RF	Random Forest
ROS	reactive oxygen species
ROC	Receiver Operating Characteristic
SHAP	Shapley Additive Explanations
SD	standard deviation
TS	training set
TcNTRs	*T. cruzi* nitroreductases
