# Supplementary material for: Dry Selection and Wet Evaluation of New 1,2-Disubstituted Nitroindazolin-3-One Derivatives as Promising Agents Against Trypanosoma cruzi
Source: Pharmaceuticals (Basel). 2026 Sep 17;19(9):1473. doi: 10.3390/ph19091473 (PMC13610718; doi:10.3390/ph19091473)
Supplement: Supplementary file 1 [file pharmaceuticals-19-01473-s001.zip › pharmaceuticals-4045504-supplementary.pdf]

Supplementary Material

Table of Contents

**Table S1:** Cathodic (Ec) and anodic (Ea) peak potentials obtained by cyclic voltammetry for compounds **1a**–**2d**, recorded at a scan rate of 2.00 V/s in DMSO using a hanging mercury drop electrode (HMDE). The table also includes the calculated half-wave potentials ($E_{1/2}$) for the observed redox couples.	**2**
**Table S2:** Hyperfine coupling constants of 1,2-disubstituted nitroindazolin-3-ones obtained by semi-empirical computation.	**3**
**Table S3:** Calculated global electronic properties (in kcal/mol) of the studied compounds. The table includes the energies of the highest occupied molecular orbital (HOMO, H) and the lowest unoccupied molecular orbital (LUMO, L), the HOMO–LUMO energy gap ($\Delta\varepsilon_{H-L}$), electron affinity (EA), ionization potential (IE), chemical hardness (η), and electrophilicity index (ω). k: Values calculated based on Koopmans' theorem / v: values obtained using the vertical approach to evaluate the electronic properties, keeping the geometry of the neutral state fixed	**4**
**Table S4:** Nucleophilic Fukui index (f^+) and spin densities ρ_{spin}(RA) of the radical anion for compounds **1a**–**1d** and **2a**–**2c**	**5**

Table S1: Cathodic (Ec) and anodic (Ea) peak potentials obtained by cyclic voltammetry for compounds **1a**–**2d**, recorded at a scan rate of 2.00 V/s in DMSO using a hanging mercury drop electrode (HMDE). The table also includes the calculated half-wave potentials ($E_{1/2}$) for the observed redox couples.

Compound	Ec-I/V	EC-II/V	EC-III/V	Ea-I/V	Ea-II/V	Ea-III/V	-E1/2/V
1a		-1.09			-1.01		1.05
2a		-1.10			-1.03		1.07
1b		-1.52			-0.59		1.06
2b		-1.50			-0.52		1.01
1c	-0.62	-0.90	-1.62	-0.58	-0.82	-1.51	0.86
2c	-0.62	-0.89	-1.50	-0.49	-0.56	-0.80	0.73
1d		-0.97			-0.81		0.89
2d		-0.95			-0.86		0.91

$E_{1/2}$: (E_{pc} + E_{pa})/2.

Table S2: Hyperfine coupling constants of 1,2-disubstituted nitroindazolin-3-ones obtained by semi-empirical computation.

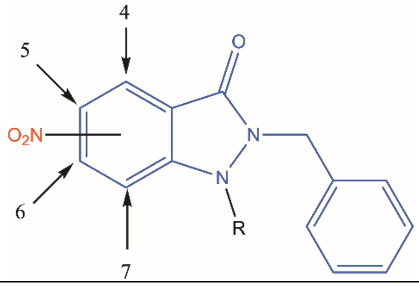

**Compounds**	**aNO$_2$**	**aN$_2$**	**aH$_4$**	**aH$_5$**	**aH$_6$**	**aH$_7$**
**1a** [19]	10.28	1.42	2.42		4.57	0.97
**2a**	10.50	1.43	2.33		4.56	1.01
**1b**	6.89			3.89	0.79	3.88
**2b**	7.01			3.91	0.92	3.90
**1c**	7.81		1.52	4.18		1.69
**2c**	7.85		1.30	4.35		1.94
**1d**	8.73		0.68	4.63	5.03	
**2d**	8.93		1.30	3.47	5.25	

Table S3: Calculated global electronic properties (in kcal/mol) of the studied compounds. The table includes the energies of the highest occupied molecular orbital (HOMO, H) and the lowest unoccupied molecular orbital (LUMO, L), the HOMO–LUMO energy gap ($\Delta\varepsilon_{H-L}$), electron affinity (EA), ionization potential (IE), chemical hardness (η), and electrophilicity index (ω). k: Values calculated based on Koopmans' theorem / v: values obtained using the vertical approach to evaluate the electronic properties, keeping the geometry of the neutral state fixed

	**1a**	**2a**	**1b**	**2b**	**1c**	**2c**	**1d**	**2d**
**H**	-178.56	-180.59	-176.83	-181.03	-178.24	-183.15	-176.37	-175.86
**L**	-32.08	-33.00	-32.31	-34.36	-42.55	-43.89	-40.01	-39.00
**$\Delta\varepsilon_{H-L}$**	146.48	147.59	144.52	146.67	135.69	139.27	136.36	136.87
**EA (k/v)**	32.08 /14.85	33.00 /16.06	32.31/15.42	34.36/18.17	**42.55/25.23**	**43.89/27.02**	40.01/22.57	39.00/22.28
**IE (k/v)**	178.56/190.84	180.59/191.66	176.83/189.34	181.03/192.42	178.24/193.15	183.15/194.40	176.37/188.75	175.86/186.94
**η (k/v)**	73.24 /87.99	73.80 /87.80	72.26/86.96	73.33/87.12	67.85/83.96	69.63/83.69	68.18/83.09	68.40/82.33
**ω (k/v)**	75.72 /60.10	77.28 /61.43	75.66 /60.27	79.08/63.63	**89.81/71.00**	**92.53/73.23**	85.84/67.18	84.32/66.46

Table S4: Nucleophilic Fukui index (f^{+}) and spin densities ρ_{spin}(RA) of the radical anion for compounds **1a–1d** and **2a–2c**

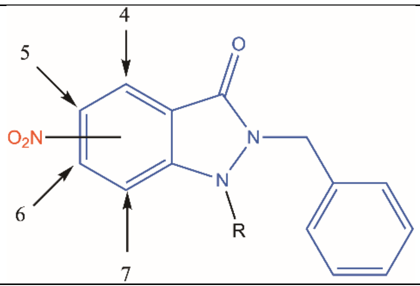

Atom No.	f^{+}/ ρ_{spin}(RA) Molecules							
	**1a**	**2a**	**1b**	**2b**	**1c**	**2c**	**1d**	**2d**
1	-0.01/0.00	-0.01/0.00	0.00/0.00	-0.01/0.01	0.00/0.00	0.00/0.00	0.00/0.00	-0.01/0.00
2	-0.01/0.00	0.00/0.00	0.02/0.00	0.01/0.00	-0.01/0.00	0.01/0.00	-0.01/0.00	-0.01/0.00
3	0.00/0.00	0.01/0.00	0.02/0.00	0.02/0.00	0.01/0.00	0.01/0.00	0.01/0.00	0.01/0.00
4	0.01/0.00	0.01/0.00	0.02/0.00	0.02/0.00	0.01/0.00	0.02/0.00	0.01/0.00	0.01/0.00
5	0.01/0.00	0.01/0.00	0.00/0.00	0.01/0.00	0.01/0.00	0.01/0.00	0.01/0.00	0.01/0.00
6	0.01/0.00	0.00/0.00	-0.01/0.00	-0.01/0.00	0.00/0.00	0.00/0.00	0.00/0.00	0.00/0.00
7	0.01/0.00	0.01/0.00	0.02/0.00	0.01/0.01	0.02/0.00	0.02/0.01	0.02/0.00	0.02/0.00
8	0.01/0.00	0.01/0.00	0.04/0.03	0.03/0.03	0.05/0.03	0.05/0.02	0.04/0.00	0.03/0.00
9	0.01/0.00	0.01/0.00	0.00/0.06	0.04/0.07	0.00/0.04	0.00/0.05	-0.01/0.03	-0.01/0.02
10	0.02/0.00	0.03/0.00	0.04/0.09	0.04/0.09	0.05/0.09	0.05/0.09	0.03/0.02	0.02/0.02
11	**0.05/0.09**	**0.06/0.11**	0.01/0.01	0.02/0.01	0.02/0.02	0.02/0.02	0.02/0.02	0.02/0.03
12	**0.03/0.03**	**0.03/0.03**	0.04/0.00	0.02/0.00	0.04/0.01	0.04/0.00	0.03/0.00	0.04/0.00
13	0.04/0.00	0.03/0.01	0.03/0.04	0.05/0.06	0.02/0.03	0.02/0.05	0.01/0.02	0.01/0.01
14	0.07/0.15	0.06/0.11	0.06/0.14	0.05/0.14	0.06/0.04	0.06/0.04	0.11/0.17	0.11/0.16

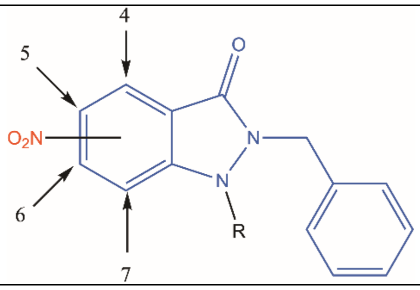

f^+/ ρ_{spin}(RA)								
**Atom No.**	**Molecules**							
	**1a**	**2a**	**1b**	**2b**	**1c**	**2c**	**1d**	**2d**
15	0.03/0.07	0.03/0.08	0.05/0.03	0.04/0.03	0.03/0.03	0.03/0.03	0.03/0.00	0.03/0.00
16	0.03/0.05	0.04/0.07	0.06/0.08	0.07/0.09	0.05/0.11	0.04/0.11	0.08/0.15	0.08/0.14
17	0.04/0.00	0.04/0.00	0.09/0.13	0.08/0.12	0.06/0.11	0.06/0.11	0.03/0.06	0.03/0.06
**18**	0.11/0.22	0.11/0.21	0.05/0.11	0.06/0.10	**0.08/0.16**	**0.08/0.15**	0.08/0.18	0.09/0.20
**19**	0.16/0.18	0.16/0.18	0.16/0.12	0.11/0.11	**0.17/0.14**	**0.16/0.13**	0.17/0.17	0.18/0.17
**20**	0.17/0.19	0.17/0.18	0.05/0.10	0.11/0.09	**0.09/0.16**	**0.08/0.16**	0.08/0.14	0.08/0.16

Note: f^+ denotes the nucleophilic Fukui index; ρ_{spin}(RA) refers to the spin density of the radical anion.

References

19. Pozo-Martínez, J.; Arán, V.J.; Zúñiga-Bustos, M.; Parra-Magna, S.; Rocha-Valderrama, E.; Liempi, A.; Castillo, C.; Olea-Azar, C.; Moncada-Basualto, M. In Vitro Evaluation of New 5-Nitroindazolin-3-one Derivatives as Promising Agents against Trypanosoma cruzi. *Int. J. Mol. Sci.* **2024**, *25*, 11107, doi:10.3390/ijms252011107.
